# Mapping the temporal and spatial dynamics of the human endometrium in vivo and in vitro

**DOI:** 10.1038/s41588-021-00972-2

**Published:** 2021-12-02

**Authors:** Luz Garcia-Alonso, Louis-François Handfield, Kenny Roberts, Konstantina Nikolakopoulou, Ridma C. Fernando, Lucy Gardner, Benjamin Woodhams, Anna Arutyunyan, Krzysztof Polanski, Regina Hoo, Carmen Sancho-Serra, Tong Li, Kwasi Kwakwa, Elizabeth Tuck, Valentina Lorenzi, Hassan Massalha, Martin Prete, Vitalii Kleshchevnikov, Aleksandra Tarkowska, Tarryn Porter, Cecilia Icoresi Mazzeo, Stijn van Dongen, Monika Dabrowska, Vasyl Vaskivskyi, Krishnaa T. Mahbubani, Jong-eun Park, Mercedes Jimenez-Linan, Lia Campos, Vladimir Yu. Kiselev, Cecilia Lindskog, Paul Ayuk, Elena Prigmore, Michael R. Stratton, Kourosh Saeb-Parsy, Ashley Moffett, Luiza Moore, Omer A. Bayraktar, Sarah A. Teichmann, Margherita Y. Turco, Roser Vento-Tormo

**Affiliations:** 1 Wellcome Sanger Institute, Cambridge, UK; 2 Centre for Trophoblast Research, University of Cambridge, Cambridge, UK; 3 Department of Pathology, University of Cambridge, Cambridge, UK; 4 EMBL-EBI, Wellcome Genome Campus, Hinxton, UK; 5 Theory of Condensed Matter Group, Cavendish Laboratory, University of Cambridge, Cambridge, UK; 6 Department of Haematology, University of Cambridge, Cambridge, UK; 7 Cambridge Biorepository for Translational Medicine (CBTM), NIHR Cambridge Biomedical Research Centre, Cambridge, UK; 8 Department of Pathology, Cambridge University Hospitals NHS Foundation Trust, Cambridge, UK; 9 Department of Immunology, Genetics and Pathology and Science for Life Laboratory, Uppsala University, Uppsala, Sweden; 10 Department of Women’s Services, Newcastle-upon-Tyne Hospitals NHS Foundation Trust, Newcastle upon Tyne, UK; 11 Department of Surgery, University of Cambridge, Cambridge, UK; 12 Present Address: Friedrich Miescher Institute for Biomedical Research, Basel, Switzerland

**Keywords:** Cell biology, Biochemistry

## Abstract

The endometrium, the mucosal lining of the uterus, undergoes dynamic changes throughout the menstrual cycle in response to ovarian hormones. We have generated dense single-cell and spatial reference maps of the human uterus and three-dimensional endometrial organoid cultures. We dissect the signaling pathways that determine cell fate of the epithelial lineages in the lumenal and glandular microenvironments. Our benchmark of the endometrial organoids reveals the pathways and cell states regulating differentiation of the secretory and ciliated lineages both in vivo and in vitro. In vitro downregulation of WNT or NOTCH pathways increases the differentiation efficiency along the secretory and ciliated lineages, respectively. We utilize our cellular maps to deconvolute bulk data from endometrial cancers and endometriotic lesions, illuminating the cell types dominating in each of these disorders. These mechanistic insights provide a platform for future development of treatments for common conditions including endometriosis and endometrial carcinoma.

## Abstract (web summary)

Single-cell and spatial transcriptomic profiling of the human endometrium highlights pathways governing the proliferative and secretory phases of the menstrual cycle. Analyses of endometrial organoids show that WNT and NOTCH signaling modulate differentiation into the secretory and ciliated epithelial lineages, respectively.

## Main

The human endometrium is the site of implantation that provides nutritional support to the placenta throughout pregnancy. Unlike other mucosal tissues, it undergoes dynamic, cyclical changes of shedding, regeneration and differentiation throughout reproductive life coordinated by the hypothalamic–pituitary–ovarian axis. Endometrial dysfunction underpins many common disorders, including abnormal uterine bleeding, infertility, miscarriage, pre-eclampsia, endometriosis and endometrial carcinoma, that collectively affect many women across the world^1–5^. Throughout reproductive years, the functional upper layer of the endometrium, the stratum functionalis, is shed at menstruation. The subsequent tissue repair and proliferation are driven by rising levels of estrogen, secreted by the ovarian follicle during the first half of the menstrual cycle (proliferative phase). Following ovulation, progesterone, produced by the corpus luteum, induces the secretory phase during which the initial changes of decidualization occur. Menstruation and spontaneous decidualization are unique to higher simian primates^6–8^. Thus, dissecting the mechanisms that regulate cellular differentiation across the menstrual cycle in humans is crucial for understanding how normal endometrium is regulated.

Essential to endometrial function are the lumenal and glandular epithelia, composed of a mixture of ciliated and secretory cells. The lumenal epithelium is the site of embryo attachment covering the endometrial surface. Long tubular glands open into the lumenal epithelium and produce secretions that are rich in growth factors and lipids necessary for placental growth^9^. We and others have established a three-dimensional in vitro organoid culture model of human endometrial epithelium^10,11^. These organoids are generated from dissociated endometrial tissue and menstrual fluid samples^12^; they retain the morphology, function and gene signature of the tissue in vivo and respond functionally to ovarian hormones with differentiation into ciliated and secretory cells. They are therefore powerful platforms to investigate endometrial disorders and study mechanisms regulating endometrial differentiation in humans. However, a systematic, quantitative comparison of endometrial organoids at a single-cell level with epithelial cell states in vivo is lacking. This is required to confirm their suitability for exploring the cellular pathways and processes involved in normal and pathological endometrial function.

The explosion in spatial transcriptomics technologies^13–16^ provides a unique opportunity to resolve tissue architecture in conjunction with underlying cellular interactions. The spatial arrangement of cells is key to understanding a morphologically complex tissue such as the endometrium, where a cell’s function may differ depending on signals it receives from neighboring cells^17^. Many spatially resolved transcriptomics methods are not quite at single-cell resolution and rely on the computational integration of coupled single-cell (or single-nucleus) transcriptomes to achieve this level of detail^18–20^. These genomic technologies are the basis of the Human Cell Atlas initiative, which aims to map all cells in the human body^21^.

In this study, by using single-cell and spatial transcriptional profiling, we interrogate the cellular states and spatial localization of human endometrial cells during the proliferative and secretory phases of the menstrual cycle in women of reproductive age. We develop CellPhoneDB v.3.0 to measure intercellular communication taking into account spatial coordinates of cells and use this tool to define cell signaling in both lumenal and glandular epithelial microenvironments. We define a complementary role for WNT and NOTCH signaling in regulating differentiation toward the two main epithelial lineages (ciliated and secretory). We profile three-dimensional endometrial organoids at single-cell resolution to characterize their hormonal responses in vitro and design a computational toolkit to compare the results with those observed in vivo to benchmark this model system. Finally, by modulating WNT and NOTCH pathways in the organoid cultures, we develop lineage-specific endometrial epithelial cells and define the molecular events involved in their response to ovarian hormones.

## Results

### A single-cell map of the full-thickness human uterus

**Fig. 1:**
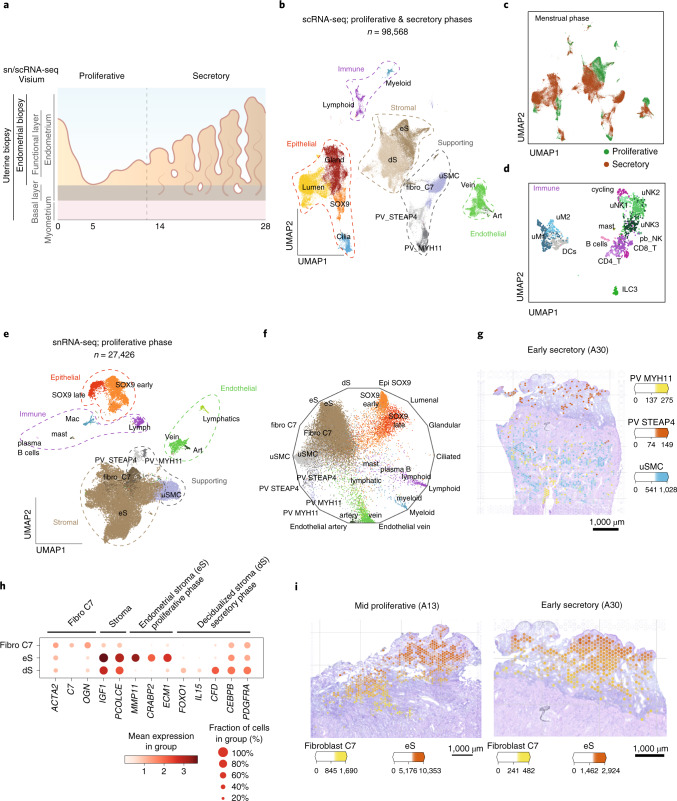
Single-cell profiling of the human uterus. **a**, Schematic illustration of the human uterus showing the different layers and the morphological changes seen throughout the menstrual cycle with respect to tissue sampling. **b**, UMAP projections of scRNA-seq data from a total of 15 individuals. **c**, UMAP representations colored by menstrual phase. **d**, UMAP of subclustered immune populations. **e**, UMAP projections of snRNA-seq data from a total of four individuals in the proliferative phase. **f**, Radial representation of the cosine distance similarity for single cells obtained from snRNA-seq to the centroids of cell types defined by scRNA-seq. **g**, Estimated amount of mRNA (color intensity) contributed by each cell population to each spot (color) shown over the hematoxylin and eosin (H&E) image of the secretory endometrium (A30, 152811 slide). **h**, Dot plot showing log_2_-transformed expression of genes expressed in fibroblast and stromal subsets. **i**, Estimated amount of mRNA (color intensity) contributed by each cell population to each spot (color) shown over the H&E image of proliferative (A30, 152810 slide) and secretory (A30, 152811 slide) endometrium. Art, artery; DC, dendritic cell; fibro, fibroblast; Gland, glandular; ILC, innate lymphoid cell; Lymph, lymphoid; Lumen, lumenal; Mac, macrophage; PV, perivascular; T, T cell; uM, uterine macrophage; uNK, uterine natural killer cell; uSMC, uterine smooth muscle cell.

**Extended Data Fig. 1:**
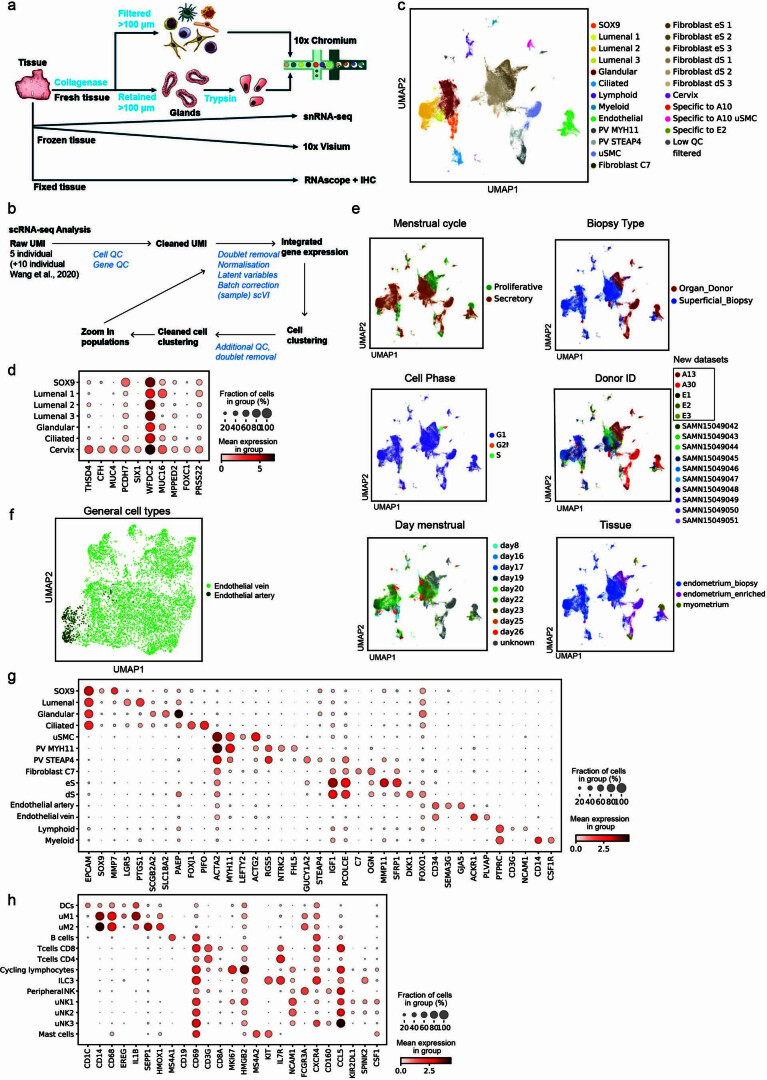
Quality control of the scRNA-seq datasets. **a**, Experimental workflow for the generation of cellular profiling of the uterus. In short, single-cell suspensions were obtained following two protocols: (i) collagenase treatment to enrich for the stromal fraction (ii) collagenase followed by trypsin to enrich for the glandular fraction. In addition, tissue blocks were processed for single-nuclei RNA sequencing (snRNA-seq) and Visium experiments. **b**, Single-cell RNA sequencing (ScRNA-seq) data analysis strategy. In short, quality control was performed at the cell and gene level on the matrices generated by STARsolo. To integrate data from distinct individuals, data was batch corrected by each sample using scVI. After defining cell clusters, those clusters containing a high proportion of low-quality cells and doublets (defined by scrublet) were excluded. Re-clustering was performed on epithelial, endothelial and immune cells. **c**, UMAP (uniform manifold approximation and projection) of scRNA-seq data from all tissue samples. Clusters corresponding to doublets, low QC cells and epithelial cells from the cervix were further excluded from the analysis. **d**, Dot plot showing log_2_-transformed expression of specific markers for the population labelled as ‘cervix’, absent in organ donor samples. Contamination from the cervix is possible due to the biopsy procedure. **e**, UMAP representations coloured by menstrual stage, biopsy type, menstrual day, tissue type, donor ID and cell cycle phase. **f**, UMAP of sub-clustered endothelial populations. **g**, Dot plot showing log_2_-transformed expression of selected genes that distinguish the main cell populations. **h**, Dot plot showing log_2_-transformed expression of selected immune cell markers. uSMC = uterine smooth muscle cell; PV = perivascular; eS = non-decidualised endometrial stromal cells; dS = decidualised endometrial stromal cells; uM = uterine macrophages; uNK = uterine Natural Killer cells, T = T cells, ILC = Innate lymphoid cells, DC = Dendritic cells; scRNA-seq = single-cell RNA sequencing, IHC = Immunohistochemistry.

To generate a cellular map of the human endometrium that accounts for the temporal and spatial changes across the menstrual cycle, uterine samples were analyzed by single-cell transcriptomics—single-cell RNA sequencing (scRNA-seq) and single-nucleus RNA sequencing (snRNA-seq)—alongside spatial transcriptomics methods (10x Genomics Visium slides and high-resolution microscopy) (Fig. 1a and Extended Data Fig. 1a). Two different types of samples from women of reproductive age were integrated in our analysis: endometrial biopsies from live donors screened for potential endometrial disorders (*n* = 3) and the whole endometrium with attached subjacent myometrium from the uteri of donors who died of nongynecological causes (*n* = 6) (Supplementary Table 1). This latter approach allows sampling of the endometrial basal layer and myometrium, which are absent from endometrial biopsies.

**Extended Data Fig. 2:**
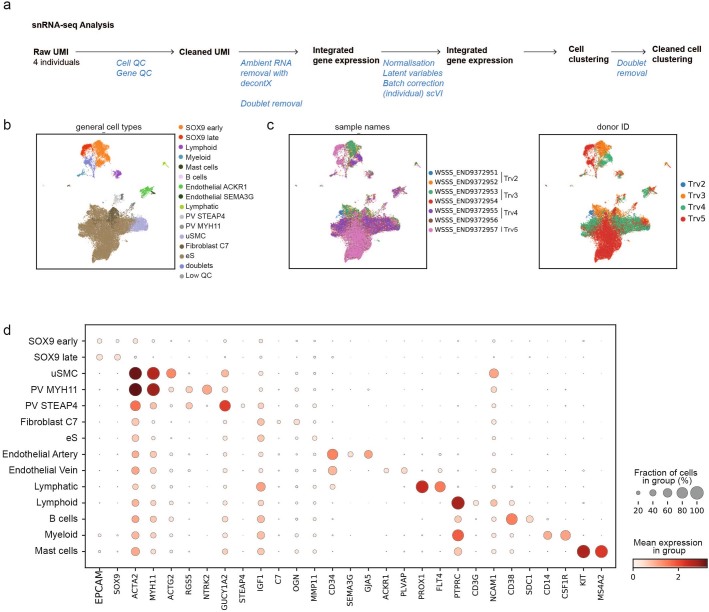
Quality control of the snRNA-seq datasets. **a**, SnRNA-seq data analysis strategy. Prior to data integration, ambient RNA was removed. **b**, UMAP (uniform manifold approximation and projection) of snRNA-seq data from all tissue samples. Clusters corresponding to doublets and low QC cells were further excluded from the analysis. **c**, UMAP representations coloured by sample ID and donor ID. **d**, Dot plot showing log_2_-transformed expression of selected genes that distinguish the main cell populations. uSMC = uterine smooth muscle cell; PV = perivascular; eS = non-decidualised endometrial stromal cells; snRNA-seq = single-nuclei RNA sequencing.

We were able to generate a uterine map of 98,568 cells from 15 individuals by integrating our dataset with previous scRNA-seq data obtained from superficial endometrial biopsies^22^ (Fig. 1b, Extended Data Fig. 1b–f and Supplementary Table 2). We identify 14 clusters that were assigned cell identity based on their expression of known markers (Extended Data Fig. 1g). These clusters can be grouped into five main cellular categories: (1) immune (lymphoid and myeloid); (2) epithelial (*SOX9*^+^, lumenal, glandular and ciliated); (3) endothelial (arterial and venous); (4) supporting—perivascular cells (PV *STEAP4* and PV *MYH11*), smooth muscle cells and fibroblasts expressing C7 (fibroblasts C7); and (5) stromal—nondecidualized endometrial (eS) and decidualized endometrial (dS). *SOX9*^+^ epithelial cells and eS are characteristic of the regenerating proliferative phase (Fig. 1c). Subclustering of immune cells resolves their heterogeneity, including identification of the three uterine natural killer cell subsets we have previously defined in the early pregnant uterus^23^ (Fig. 1d and Extended Data Fig. 1h). snRNA-seq data from four additional full-thickness uterine samples of proliferative endometrium confirm the populations found in the proliferative phase (Fig. 1e,f and Extended Data Fig. 2a–d). We additionally found lymphatic endothelial and mast cells enriched in these samples; these are likely to originate from the myometrium (Fig. 1e and Extended Data Fig. 2d).

**Extended Data Fig. 3:**
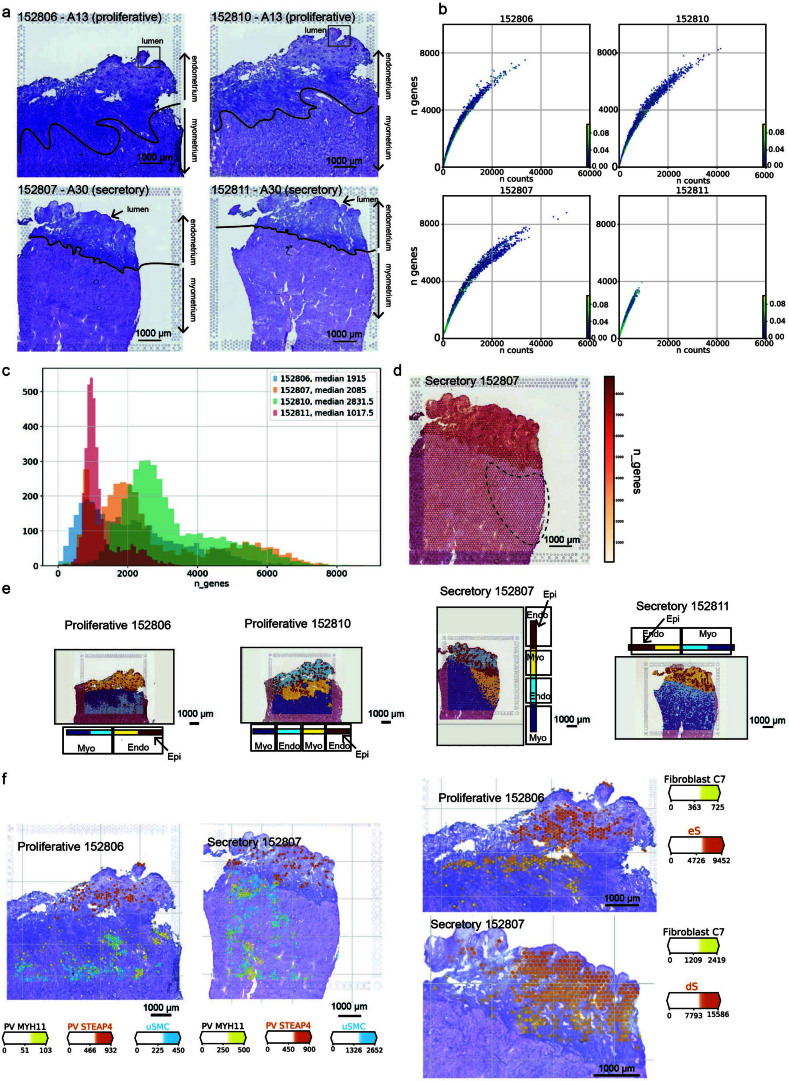
Quality control of the Visium slides. **a**, Haematoxylin and eosin staining of the slides in the Visium arrays. Two individuals were selected: proliferative phase A13 and secretory phase A30. Two sections 100 μm apart were analysed. Lumenal epithelium was well preserved in individual A30 and in a small region of A13. **b**, Scatter plots show the number of genes over the number of counts, where each dot is a feature of the Visium slide. Plots are coloured by the percentage of mitochondrial genes. **c**, Bar plots showing number of genes on each of the samples. A bimodal distribution corresponding to endometrium and myometrium was shown on each of the cases. **d**, Visualisation of the number of genes on the Visium slides of sample A30, 152807 slide. A zone with low quality is highlighted in the image. This is probably caused by a technical artifact. No pattern like this was seen in other samples. **e**, Unbiased clustering of Visium spots defined by Louvain algorithm. **f**, Estimated amount of mRNA (colour intensity) contributed by each cell population to each spot (colour) shown over the H&E image of the proliferative (A13, 152806 slide) and secretory (A30, 152807 slide) endometrium. Endo = Endometrium; Myo = Myometrium; Epi = Epithelial; uSMC= uterine smooth muscle cell; PV = perivascular.

To systematically map the location of the cell types identified by scRNA-seq within the endometrium and myometrium, we used Visium Spatial Transcriptomics technology. We examined four full-thickness uterine samples in the proliferative and secretory phases from two individuals (Extended Data Fig. 3a–e). After integrating single-cell transcriptomics and Visium data using our recently developed cell2location algorithm^18^, cell states were mapped to the endometrium and/or the myometrium. We identify specific perivascular cells: PV *MYH11* are characteristic of myometrium while PV *STEAP4* are only present in the endometrium (Fig. 1g and Extended Data Fig. 3f). In addition, we find that fibroblasts C7 are enriched in the basal layer of the endometrium in both the proliferative and secretory phases (Fig. 1h,i and Extended Data Fig. 3f).

Altogether, our analysis yields a comprehensive catalog of the major subsets of uterine cells together with their cellular position in endometrium and myometrium. We have made an open-source web server available at www.reproductivecellatlas.org.

### Spatiotemporal characterization of proliferative epithelium

**Fig. 2:**
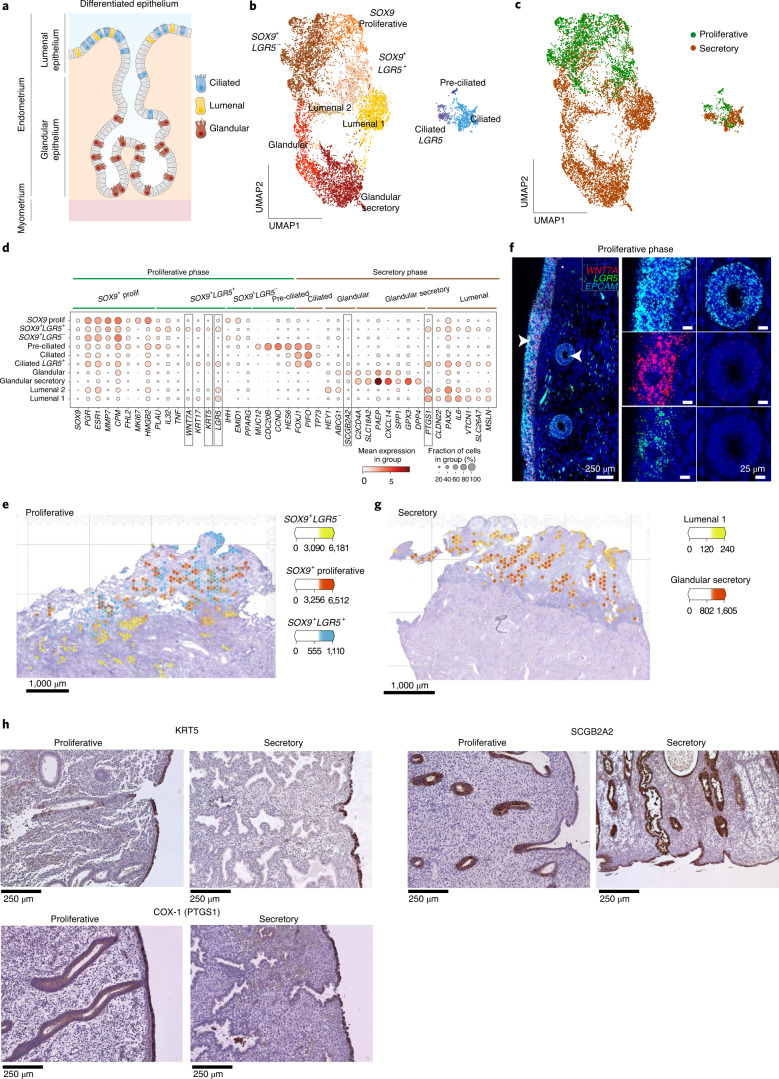
Temporal and spatial dynamics of endometrial epithelial cells. **a**, Schematic illustration of epithelial subsets in the differentiated endometrium highlighting the anatomical location of the glandular and lumenal epithelia. **b**, UMAP of subclustered and subsampled epithelial populations. **c**, UMAP of subclustered and subsampled epithelial populations colored by their menstrual phase. **d**, Dot plot showing the log_2_-transformed expression of genes characteristic of each epithelial subset. **e**, Number of mRNA molecules per spot (color intensity) confidently assigned to each epithelial subpopulation (color) in the proliferative phase (A13, 152810 slide). **f**, High-resolution large-area imaging of a section of proliferative endometrium, stained with in situ hybridization (smFISH) for *WNT7A* and *LGR5* (*SOX9*^+^*LGR5*^+^ epithelial markers). White arrowheads indicate lumenal and glandular regions shown at higher magnification (right). Representative image of four proliferative endometrial samples from four different donors. Scale bars: left, 250 μm; other, 25 μm. **g**, Number of mRNA molecules per spot (color intensity) confidently assigned to each epithelial subpopulation (color) in the early-proliferative phase (A30, 152807 slide). **h**, Validation of KRT5, COX1 (marker of lumenal cells) and SCGB2A2 (marker of glandular population) with IHC in endometrial tissue (proliferative and secretory phases). Nuclei are counterstained with hematoxylin. Scale bars, 250 μm. Representative images of three proliferative and three secretory endometrial samples from six different donors.

**Extended Data Fig. 4:**
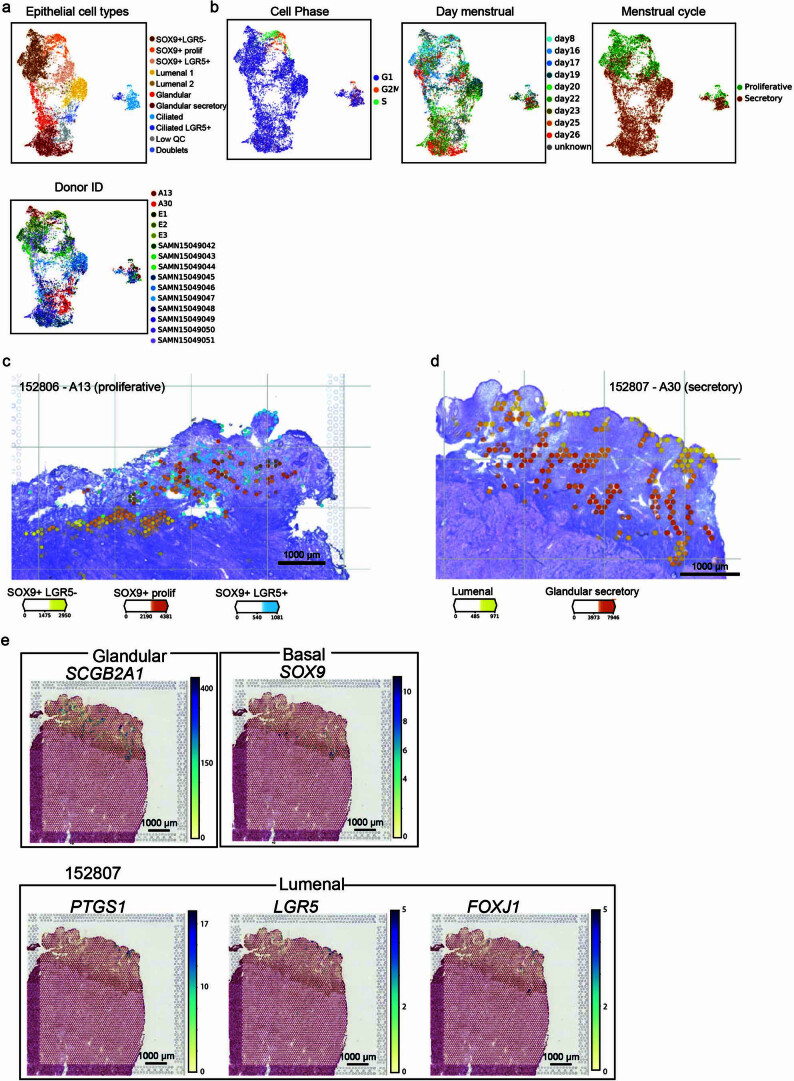
Spatio-temporal regulation of epithelial cells. **a**, UMAP (uniform manifold approximation and projection) of scRNA-seq data from epithelial cells. We performed a donor-balanced subsampling (1000 cells maximum for each donor). Clusters corresponding to doublets and low QC cells were further excluded from the analysis. **b**, UMAP representations coloured assigned by cell phase, donor, menstrual stage and day of the menstrual cycle. **c**, Number of mRNA molecules per spot (colour intensity) confidently assigned to each epithelial subpopulation (colour) in the proliferative phase (A13, 152806 slide). **d**, Number of mRNA molecules per spot (colour intensity) confidently assigned to each epithelial subpopulation (colour) in the secretory phase (A30, 152807 slide). **e**, Estimated proportion of mRNA coming from epithelial subsets in the early-proliferative phase (A30, 152807 slide).

We next focused on the two main lineages of endometrial epithelial cells, secretory and ciliated, across the menstrual cycle and analyzed these subsets individually (Fig. 2a). Epithelial cells are classified into four main groups based on their marker expression: (1) *SOX9* populations, enriched in the proliferative phase and expressing genes characteristic of rising estrogen levels (*MMP7*, *ESR1*); (2) ciliated cells (*PIFO*, *TPPP3*); (3) lumenal cells (*LGR5*); and (4) glandular cells (*SCGB2A2*) (Fig. 2b–d and Extended Data Fig. 4a,b). A fraction of the glandular epithelial cells express molecules characteristic of uterine milk in the secretory stage (*PAEP*, *CXCL8*).

**Extended Data Fig. 5:**
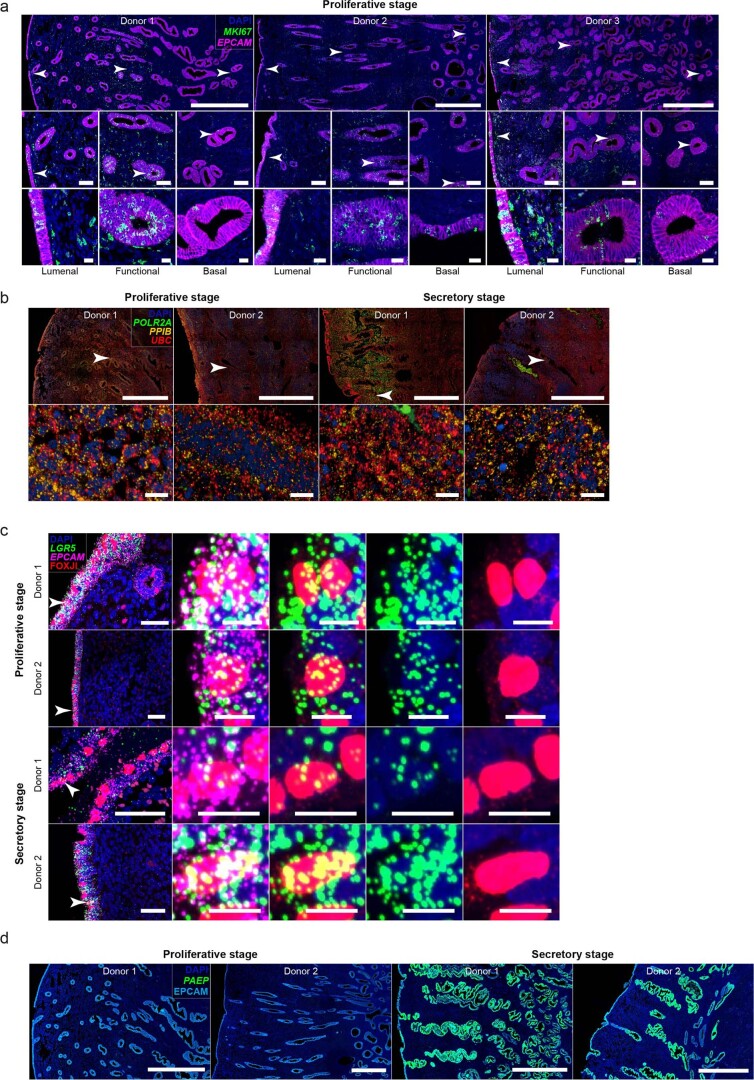
Spatially-resolved single-cell transcriptomic expression of proliferative markers by smFISH. **a**, High-resolution large-area imaging of uterine tissue sections from three donors in the proliferative phase stained with smFISH for *MKI67*, combined with protein staining for EPCAM. White arrowheads indicate magnified regions demonstrating representative *MKI67* expression levels across lumenal, functional, and basal epithelia. Scale bars, top = 1 mm, middle = 100 μm, bottom = 20 μm. **b**, Molecular integrity of uterine tissues was validated by multiplexed smFISH staining of sections for constitutively expressed genes of different typical expression levels (*UBC* - high; *PPIB* - moderate; *POLR2A* - low), which demonstrated strong signals irrespective of sample or region. White arrowheads indicate epithelial regions shown at higher magnification (bottom). Scale bars, top = 1 mm; below = 20 μm. Two representative donors each from the proliferative and secretory phases are shown. **c**, High-resolution imaging of uterine tissue sections stained with smFISH for *EPCAM* and *LGR5*, combined with protein staining of FOXJ1. White arrowheads indicate cells with dual FOXJ1 and *LGR5* staining, shown magnified to the eight. Scale bars, left = 50 μm, other = 10 μm. **d**, High-resolution large-area imaging of four endometrial sections stained with *PAEP*. Scale bars = 1 mm.

With our integrative scRNA-seq maps, we can now resolve three clusters within the *SOX9* population: (1) *SOX9*^+^*LGR5*^+^ cells, expressing *KRT17* and *WNT7A*; (2) *SOX9*^+^*LGR5*^−^ cells, expressing *IHH*; and (3) proliferative *SOX9*^+^ cells, including both *LGR5*^+^ and *LGR5*^−^ cycling cells (that is, cells in G2M/S phase). By integrating scRNA-seq and Visium data (Fig. 2e and Extended Data Fig. 4c), we define specific spatial coordinates for these *SOX9* subsets: (1) noncycling *SOX9*^+^*LGR5*^+^ cells are enriched in the surface epithelium; (2) noncycling *SOX9*^+^*LGR5*^−^ cells are located in the basal glands; and (3) cycling *SOX9*^+^ cells map to glands in the regenerating superficial layer. Single-molecule fluorescence in situ hybridization (smFISH) with RNAscope probes shows higher expression of the proliferative marker *MKI67* in the superficial layer of the endometrium during the proliferative phase, validating Visium data (Extended Data Fig. 5a). We also validated the presence of the markers *LGR5* and *WNT7A*, characteristic of the *SOX9*^+^*LGR5*^+^ cells present in surface epithelium during the proliferative phase (Fig. 2f and Extended Data Fig. 5b,c). *SOX9* and *LGR5* are expressed in stem cells/progenitors in several tissues including gut, kidney, skin and ovaries and may label similar populations in the human endometrium^24–29^.

Ciliated cells are present in both the proliferative and secretory phases, but, as expected, *PAEP* secretory cells are only present following ovulation (Fig. 2b,c and Extended Data Fig. 4b). This indicates that estrogen alone can induce ciliary differentiation, while secretory differentiation depends on the addition of progesterone. During the proliferative phase, in addition to the *FOXJ1*, *PIFO*, *TP73* ciliated population, we define a distinct subset of preciliated cells that express *MUC12*, *HES6* and cell cycle genes (*CDC20B*, *CCNO*) (Fig. 2d).

After ovulation, secretion of progesterone induces the differentiation of *SOX9*^+^ cells into specialized secretory cells found at the surface and in glands (Extended Data Fig. 5d). To date, it has not been possible to distinguish between differentiated lumenal and glandular subsets using specific transcriptomic signatures and markers. We can now identify these subsets in our scRNA-seq data, confirmed by integration with spatial transcriptomic data (Fig. 2g and Extended Data Fig. 4d,e) and revealing markers that are validated at the protein level (Fig. 2h). There is enriched expression of both COX1 (encoded by *PTGS1*) and KRT5 in the lumenal epithelium and SCGB2A2 in the glandular epithelium.

### *SOX9*^+^ epithelial cells in endometrial disorders

Disorders in endometrial function have a profound impact on women’s health and reproductive outcomes. There has been limited progress in the study of these disorders over the past decade, partly due to the challenges in analyzing this highly dynamic and complex tissue. To identify the cells involved in these disorders, we looked for transcriptomic signatures in bulk RNA data from endometrial cancers and endometriotic lesions.

**Fig. 3:**
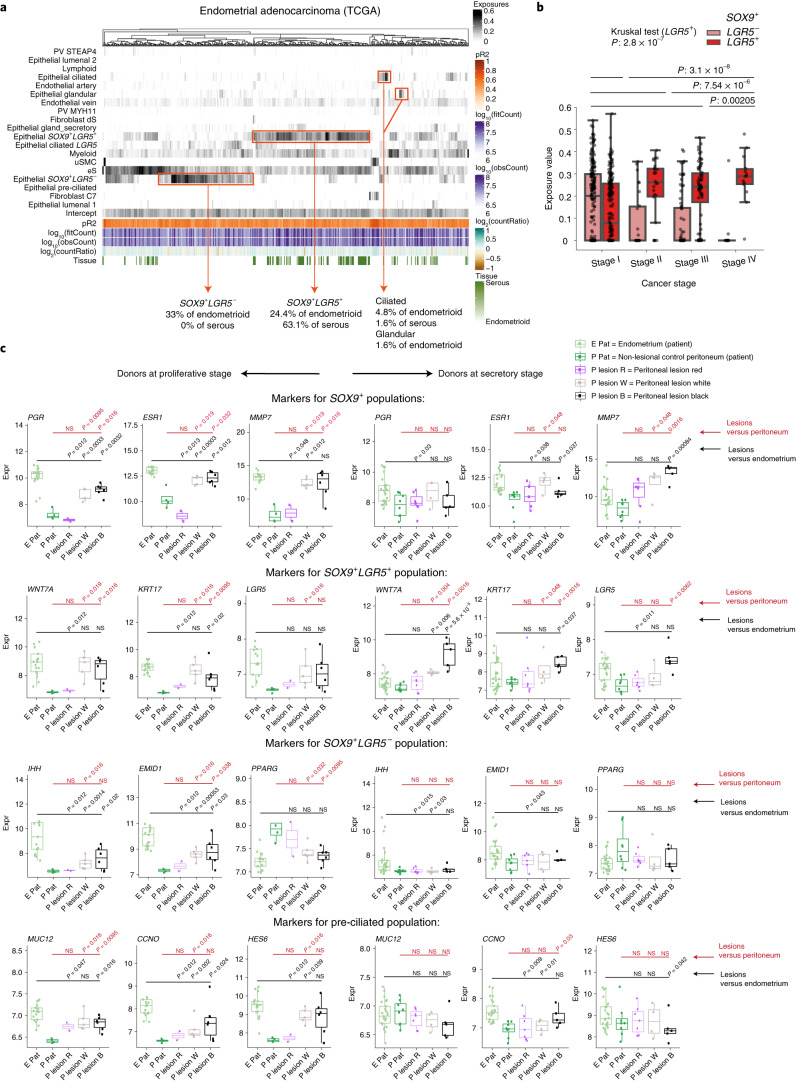
Epithelial signatures in endometrial disorders. **a**, Heatmaps showing the relative contribution of single-cell-derived signals from healthy endometrium (rows) in explaining the bulk transcriptomes of 430 endometrioid and 122 serous endometrial adenocarcinomas from TCGA (columns). **b**, Analysis of the 313 endometrial adenocarcinoma TCGA samples that exhibit *SOX9*^+^*LGR5*^+^ or *SOX9*^+^*LGR5*^−^ exposure above the intercept value. A Kruskal–Wallis test was performed on cohorts of samples regrouped in association with cancer stages from I to IV (*n* = 201, 26, 72 and 14, respectively). The *SOX9*^+^*LGR5*^+^ exposure depends on the stage of the tumor. Wilcoxon tests show that the increased value associated with later stages is significant for each stage partition (horizontal lines denote binary partitions: *n* = 201 versus 112, *n* = 227 versus 86, *n* = 299 versus 14). Black dots are individual exposure values. Boxplots represent quartiles while whiskers extend up to 1.5 times interquartile range (IQR) beyond each box to encapsulate extrema. **c**, Boxplots showing normalized expression levels of epithelial marker genes in endometrium and peritoneum from control donors and patients with endometriosis from GSE141549. Expression in red, white or black peritoneal lesions is compared with endometrium and normal peritoneum by two-sided Wilcoxon test (not significant (NS): *P* > 0.05). Boxplots represent quartiles and whiskers extend up to 1.5 times IQR beyond each box to encapsulate extrema. For the proliferative and secretory comparisons, the number of independent biological samples was, respectively: control endometrium (*n* = 17 and *n* = 25), control peritoneum (*n* = 4 and *n* = 8), peritoneum red lesions (*n* = 2 and *n* = 7), peritoneum white lesions (*n* = 5 and *n* = 4) and peritoneum black lesions (*n* = 6 and *n* = 5). E, endometrium; E Pat, endometrium (patient); Expr, expression; P, peritoneum; P lesion B, peritoneal lesion black; P lesion R, peritoneal lesion red; P lesion W, peritoneal lesion white; P Pat, nonlesional control peritoneum (patient).

**Extended Data Fig. 6:**
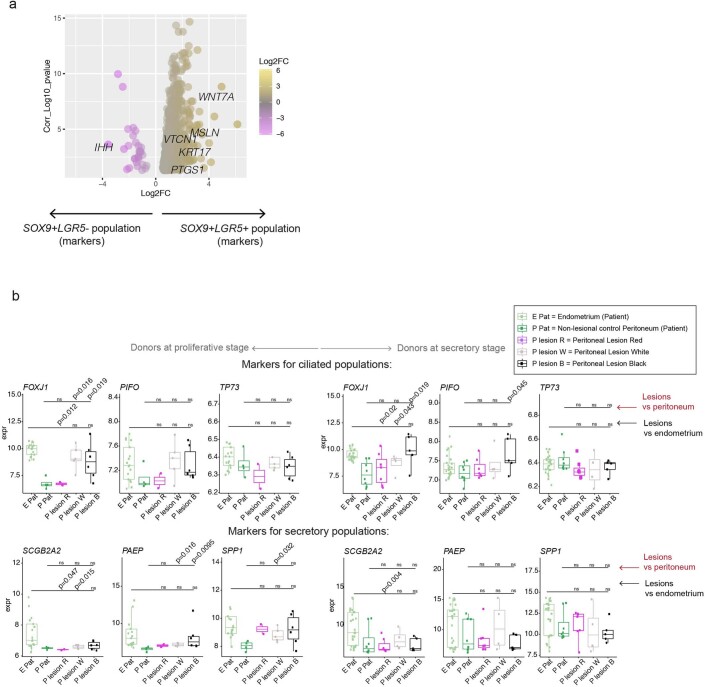
Expression of epithelial markers in endometrial disorders. **a**, Volcano plot showing upregulation of markers specific for *SOX9* + *LGR5* + in endometrial tumours with a SOX9 + LGR5 + signature in comparison with those with a *SOX9* + *LGR5-* signature. Negative log fold changes similarly show the opposite pattern where markers are characteristic of the *SOX9* + *LGR5-* population instead. **b**, Boxplots showing expression levels of epithelial marker genes in endometrium and peritoneum from patients with endometriosis from GSE141549. Expression in peritoneal lesions is compared to endometrium and peritoneum with two-sided Wilcox test (ns; not significant: p > 0.05). Box plots represent quartiles and whiskers extend up to 1.5 IQR beyond each box to encapsulate extrema. For the proliferative and secretory comparisons, the number of independent biological samples was, respectively: control endometrium (n = 17 and n = 25), control peritoneum (n = 4 and n = 8), peritoneal red lesions (n = 2 and n = 7), peritoneal white lesions (n = 5 and n = 4) and peritoneal black lesions (n = 6 and n = 5). E Pat = endometrium (patient); P Pat = non-lesional control Peritoneum (Patient); P lesion R = Peritoneal Lesion Red; P lesion W = Peritoneal Lesion White; P lesion B = Peritoneal Lesion Black.

We first deconvoluted the transcriptomes from bulk RNA sequencing data from serous and endometrioid endometrial adenocarcinomas available in The Cancer Genome Atlas (TCGA), using our single-cell atlas as a reference. Deconvolution reveals that the signature of the *SOX9*^+^ epithelial population is dominant in tumors (Fig. 3a). Based on expression signals in epithelial cells, endometrial adenocarcinomas resolve into three patterns. The first, characterized by *SOX9*^+^*LGR5*^+^, occurs in 63% of serous and 24% of endometrioid adenocarcinomas. The second, *SOX9*^+^*LGR5*^−^, is found in 33% of endometrioid adenocarcinomas but is absent from serous adenocarcinomas. The third pattern, with expression of differentiated ciliated or glandular signals, is present in <10% of endometrial carcinomas. In line with these results, markers characteristic of the *SOX9*^+^*LGR5*^+^ subset (for example, *WNT7A*, *MSLN*, *KRT17*, *PTGS1* and *VTCN1*) are all upregulated in tumors with a *SOX9*^+^*LGR5*^+^ transcriptomic signature compared to tumors with a *SOX9*^+^*LGR5*^−^ signature (Extended Data Fig. 6a).

We then correlated the clinical stages of endometrial adenocarcinomas with our cell signals. The more advanced stages of endometrial adenocarcinomas (stages III and IV) have a greater *SOX9*^+^*LGR5*^+^ signal (Wilcoxon test, stages I + II against stages III + IV, *P* value 7.54 × 10^−6^) (Fig. 3b and Supplementary Table 3). We also linked the *SOX9*^+^*LGR5*^+^ signals to the four molecular subtypes of endometrial cancer defined by TCGA^30^. The *SOX9*^+^*LGR5*^+^ signature is stronger in tumors characterized by high copy number (copy-number-high) alterations (Wilcoxon text, *P* value 6.72 × 10^−13^), typical of serous endometrial adenocarcinomas and linked with a worse prognosis (Supplementary Table 3). Indeed, all the copy-number-high tumors considered in our analysis (31 serous and 7 serous-like endometrioid adenocarcinomas) were classified as *SOX9*^+^*LGR5*^+^. In contrast, copy-number-low tumors have a lower *SOX9*^+^*LGR5*^+^ signal (Wilcoxon text, *P* value 5.91 × 10^−6^) (Supplementary Table 3).

We then explored the expression of specific epithelial markers in microarray expression data available from peritoneal biopsies from donors with endometriosis^31^ (Supplementary Table 3). As expected, endometriotic peritoneal lesions upregulate markers characteristic of proliferative endometrium (*SOX9*^+^ and preciliated markers) compared with normal peritoneum (Fig. 3c). In particular, peritoneal lesions upregulate markers specific for the *SOX9*^+^*LGR5*^+^ subset (such as *WNT7A* and *KRT17)* with expression levels similar to those in proliferative endometrium (Fig. 3c). In contrast, markers of secretory epithelial cells, *PAEP* and *SCGB2A2*, or ciliated cells, *PIFO* and *TP73*, are expressed at similar levels as in normal peritoneum (Extended Data Fig. 6b).

In summary, our analysis suggests that dysfunctional epithelium is a major driver of endometrial disease. By defining the transcriptomes of our two *SOX9* populations, we can show the specific cell signals that dominate in endometrial carcinomas and endometriosis.

### Effect of microenvironments on epithelial identity

**Fig. 4:**
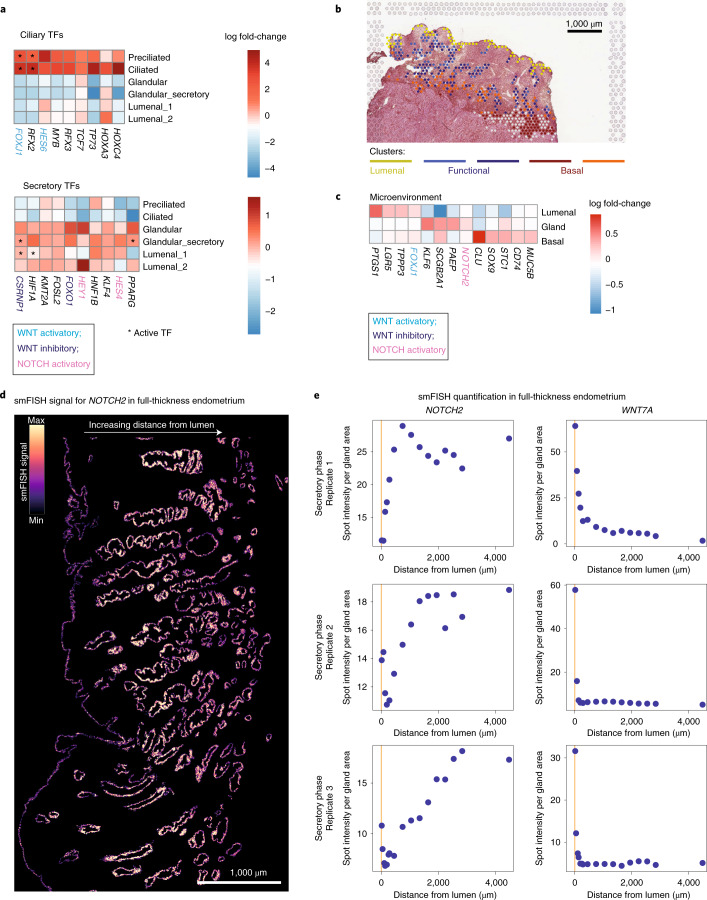
Cell signaling in glandular and lumenal epithelium. **a**, Heatmaps showing TFs differentially expressed in ciliated (top) and secretory (bottom) epithelial lineages. Color is proportional to log-transformed fold change; asterisks highlight TFs whose targets are also differentially expressed (that is, differentially activated TFs). **b**, Unbiased clustering of epithelial subsets using Visium data. Spot colors represent cluster assignment based on Louvain clustering of spots assigned to epithelial subsets. Spots assigned to one of the clusters (represented in light gray in the figure) were excluded from the analysis due to the low percentage of epithelial cells in the spot after visual inspection. **c**, Heatmap showing log-transformed fold change of differentially expressed genes between the three main clusters defining the lumenal, functional and basal epithelial regions. **d**, High-resolution large-area imaging of a representative section of secretory-phase endometrium, with pseudocolor intensity proportional to smFISH signal for *NOTCH2*. Representative image of three endometrial samples from three different donors. **e**, smFISH quantification in three full-thickness secretory-phase endometrial samples. Plots represent smFISH spot intensity in glands divided by gland area at increasing distances from the lumen. Approximate lumen range is marked in yellow. Source data

Having defined these distinct epithelial cell states and their potential role in pathology, we focused next on the transcription factors (TFs) that regulate epithelial differentiation throughout the menstrual cycle by analyzing expression of TFs and their consensus target genes^32^ (Fig. 4a and Supplementary Table 4). There is high activity of WNT targets (for example, *FOXJ1*) in the ciliated epithelium. In contrast, the glandular subsets show high expression for TFs induced by WNT inhibition (for example, *CSRNP1* and *FOXO1*) and NOTCH activation (for example, *HES1* and *HEY1*). This suggests different roles for NOTCH and WNT in shaping the identity and function of ciliated versus secretory cells.

**Extended Data Fig. 7:**
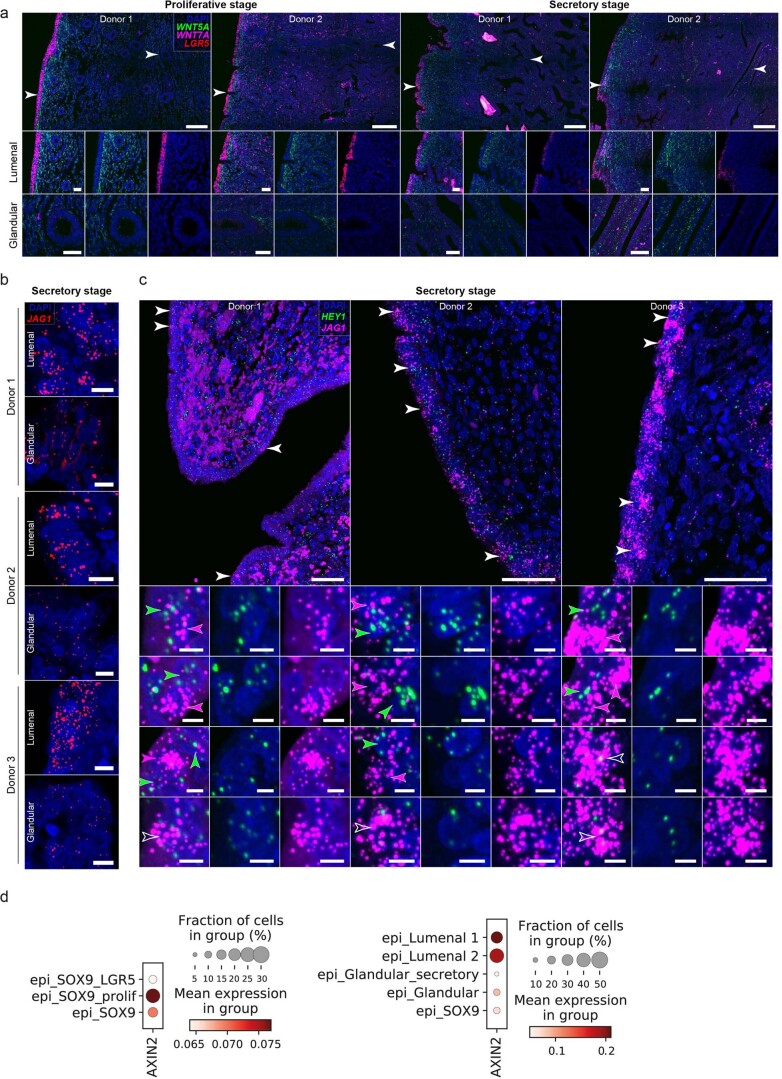
Spatially-resolved single-cell transcriptomic expression of WNT and NOTCH signals. **a**, High-resolution large-area imaging of uterine tissue sections stained with smFISH for *WNT7A*, *WNT5A*, and *LGR5*. White arrowheads indicate magnified regions demonstrating spatial segregation of *WNT7A* (lumenal epithelial) and *WNT5A* (stromal) expression. Scale bars, top = 200 μm, other = 50 μm. **b**, High-resolution imaging of endometrial tissue sections stained with smFISH for *JAG1*. A comparison of representative regions of lumenal and glandular epithelium is shown for three secretory phase donors. Scale bars = 10 μm. **c**, Co-staining of *JAG1* and *HEY1*. Top, solid white arrowheads indicate regions of lumenal epithelium magnified below. Below, cells showing juxtacrine expression of *JAG1* and *HEY1* (magenta arrowheads = *JAG1*^high^*HEY1*^low^, green arrowheads = *JAG1*^low^*HEY1*^high^) or co-expression (white outlined arrowheads). Scale bars, top = 50 μm, other = 5 μm. **d**, Dot plot showing log_2_-transformed expression of *AXIN2* expression.

To investigate the cell signals operating in the lumenal and glandular microenvironments that could influence differentiation into ciliated and secretory lineages, we used spatial transcriptomics and performed clustering on the 10x Genomics Visium spots assigned to epithelial subsets. We resolve five clusters corresponding to cells in the lumenal (one cluster), functional (two clusters) and basal (two clusters) layers (Fig. 4b). In addition to cell-type-specific markers, signatures of WNT and NOTCH signaling pathways are present in distinct endometrial regions (Fig. 4c and Supplementary Table 5). Genes involved in the WNT pathway, *FOXJ1* and *LGR5*, are highly expressed at the lumenal surface while *NOTCH2* is enriched in glands in the functional layer. To validate expression of *NOTCH2* and *WNT7A* in these compartments, we stained uterine tissue with smFISH probes for both genes alongside EPCAM using immunohistochemistry (IHC) (Fig. 4d,e). Lumenal and glandular epithelial cells were classified automatically based on EPCAM expression, and the distance of the signal from the lumen was then measured (Methods). Our results show that *NOTCH2* expression increases in glands moving away from the lumen while *WNT7A* expression is higher in the lumenal epithelium compared with glands (Fig. 4e). By contrast, the noncanonical WNT molecule *WNT5A* was mainly expressed in stromal cells surrounding the glands (Extended Data Fig. 7a). These findings suggest that canonical WNT is downregulated in the glandular microenvironment where noncanonical WNT pathways dominate.

**Fig. 5:**
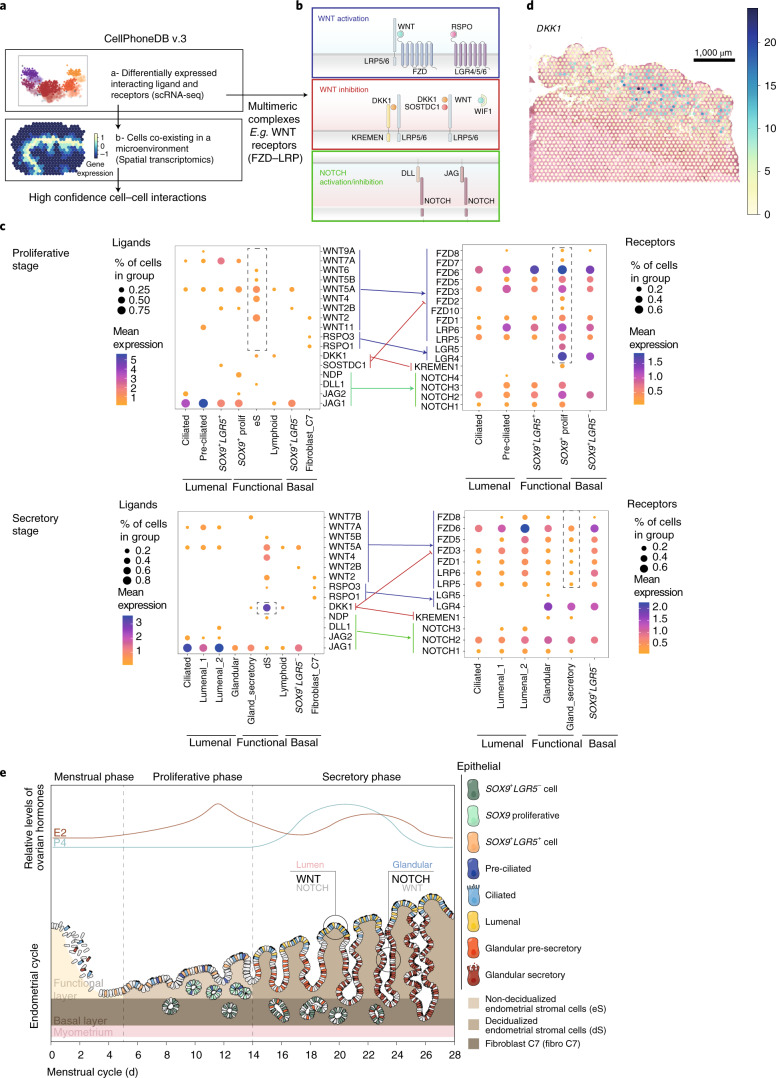
Interrogation by CellPhoneDB v.3.0 of ligands and receptors mediating epithelial differentiation. **a**, Adaptation of our cell–cell communication tool that considers spatial cellular dynamics and is available at https://github.com/Ventolab/CellphoneDB. **b**, Schematic illustration of receptors and ligands involved in WNT and NOTCH signaling. **c**, Dot plots showing expression of CellPhoneDB v.3.0 relevant ligands in epithelial, stromal and fibroblast populations with cognate receptors in epithelial subsets. Only significant interactions (fold change > 0.02 and FDR < 0.005) are represented. The color of the arrows corresponds to the pathways whose ligand–receptor partners are involved, as shown in **b**. **d**, Estimated proportions of *DKK1* in the early-proliferative phase (A30, 152807 slide). **e**, Schematic illustration of our proposed model for temporal and spatial distribution of epithelial and stromal subsets across the menstrual cycle. The proliferative phase is dominated by a WNT environment that promotes regeneration. Compartmentalization of WNT and NOTCH signaling during the secretory phase promotes efficient differentiation toward the ciliated and secretory lineages.

To investigate how surrounding cells may shape signaling in the surface and glandular compartments, we developed CellPhoneDB v.3.0, an updated version of our cell–cell communication pipeline that takes into account spatial cellular colocalization when mapping ligand–receptor pairs^33^ (Methods and Fig. 5a). CellPhoneDB considers the multimeric composition of the majority of ligands and receptors, which is highly relevant for the complex regulation of WNT signaling (Fig. 5b). We define three endometrial microenvironments centered on epithelial cells based on the cellular coordinates provided by cell2location: (1) lumenal—preciliated, ciliated and *SOX9*^+^*LGR5*^+^ epithelium (proliferative phase) and ciliated and lumenal (secretory phase); (2) functional—*SOX9*^+^ proliferative epithelium, immune and eS (proliferative phase) and immune, glandular and dS (secretory phase); and (3) basal—*SOX9*^+^*LGR5*^−^ and fibroblasts C7. We ran CellPhoneDB on each of the microenvironments (Supplementary Table 6) and found significant epithelial–stromal interactions (log fold change > 0.02 and false discovery rate (FDR) < 0.005).

NOTCH interactions are mainly mediated by the epithelial compartment (Fig. 5c), with the NOTCH ligand *JAG1* more highly expressed in the lumen than in glands, as shown by smFISH (Extended Data Fig. 7b,c). As well as *JAG1* being coexpressed with *HEY1*, sparse *JAG1*^high^ epithelial cells are often adjacent to *JAG1*^low^ cells expressing *HEY1* (Extended Data Fig. 7c). A similar lateral NOTCH inhibition model has been described in the gut^34^.

WNT ligands are expressed by both epithelial and stromal cells (Fig. 5c); the latter express WNT agonists that can potentially bind the cognate WNT receptors expressed by all epithelial subsets during the proliferative phase (Fig. 5c). Focusing on genes that are differentially expressed following ovulation, glandular secretory subsets show a dramatic decline in WNT receptor expression, potentially limiting the activity of this pathway (Fig. 5c). They also show a decrease in the WNT target *AXIN2* when compared with their lumenal counterparts (Extended Data Fig. 7d). In addition, decidualized stromal cells express significantly higher levels of *DKK1*, a potent inhibitor of the WNT pathway, than their nondecidualized counterparts. Expression of *DKK1* surrounding the glands of the secretory endothelium is also found in spatial transcriptomics (Fig. 5d). Overall, these findings strongly suggest that WNT signaling is inhibited in the secretory cell lineages, meaning that NOTCH signaling will then dominate (Fig. 5e).

### Response of endometrial organoids to ovarian hormones

**Fig. 6:**
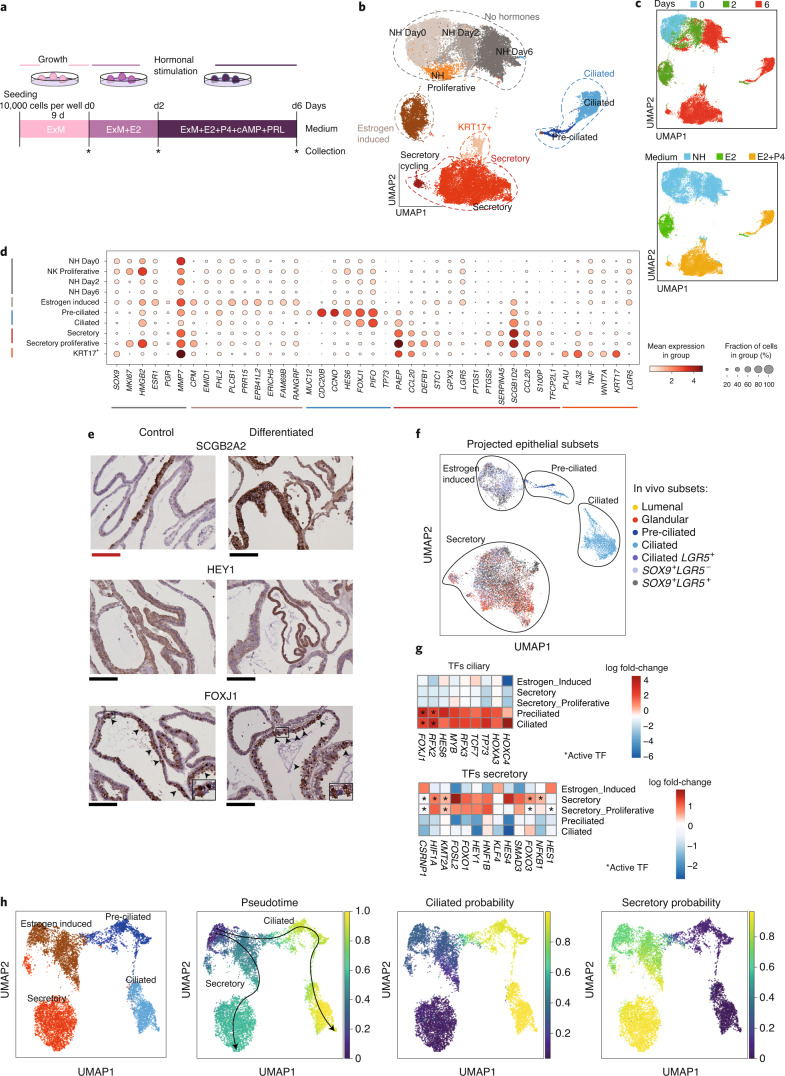
In vitro responses of endometrial organoids to ovarian hormones are similar to in vivo epithelial changes. **a**, Experimental timeline of endometrial organoid cultures. Organoids were derived in ExM and then subjected to hormonal stimulation with estrogen (E2) followed by estrogen + progesterone (P4) + cAMP and prolactin (PRL). The time points at which organoids were collected for scRNA-seq are marked with an asterisk. **b**, UMAP projections of scRNA-seq data identify major cellular populations. **c**, UMAP representations colored by days after hormonal stimulation (top) or by treatments (bottom). **d**, Dot plot showing log_2_-transformed expression of selected genes that distinguish the main cell populations. **e**, IHC to validate markers of the secretory population, SCGB2A2 and HEY1, and combined staining for FOXJ1 and acetylated α-tubulin in control (undifferentiated) and differentiated (hormonally stimulated) organoids. Black arrowheads indicate ciliated cells with FOXJ1-positive nuclei. Nuclei are counterstained with hematoxylin. Scale bars: 250 μm (red), 200 μm (black). Representative images of three endometrial organoids from three different donors. **f**, Predicted epithelial subsets of endometrial organoids using a logistic classifier. **g**, Heatmaps showing TFs differentially expressed in ciliated and secretory lineages. Color is proportional to log-transformed fold change, with asterisks highlighting TFs whose targets are also differentially expressed (that is, differentially activated TFs). **h**, Cells able to respond to progesterone derived from a clonal organoid culture (E001 individual) (Methods) are colored from left to right: (1) cluster labels as in Extended Data Fig. 8e; (2) Palantir pseudotime; (3) probability of cells to progress toward the ciliary lineage; and (4) probability that the cell differentiates toward the secretory lineage. NH, no hormone.

To test our predictions on the potential roles of WNT and NOTCH signaling pathways on endometrial epithelium in vitro, we first profiled endometrial organoids at a single-cell level to benchmark this model system against our in vivo data. We derived organoids^10^ from three different donors and primed them as previously described with estrogen for 48 h, followed by stimulation with progesterone, prolactin and cyclic AMP (cAMP) in the presence of estrogen for 4 d (ref. ^10^) (Fig. 6a and Supplementary Tables 7 and 8).

**Extended Data Fig. 8:**
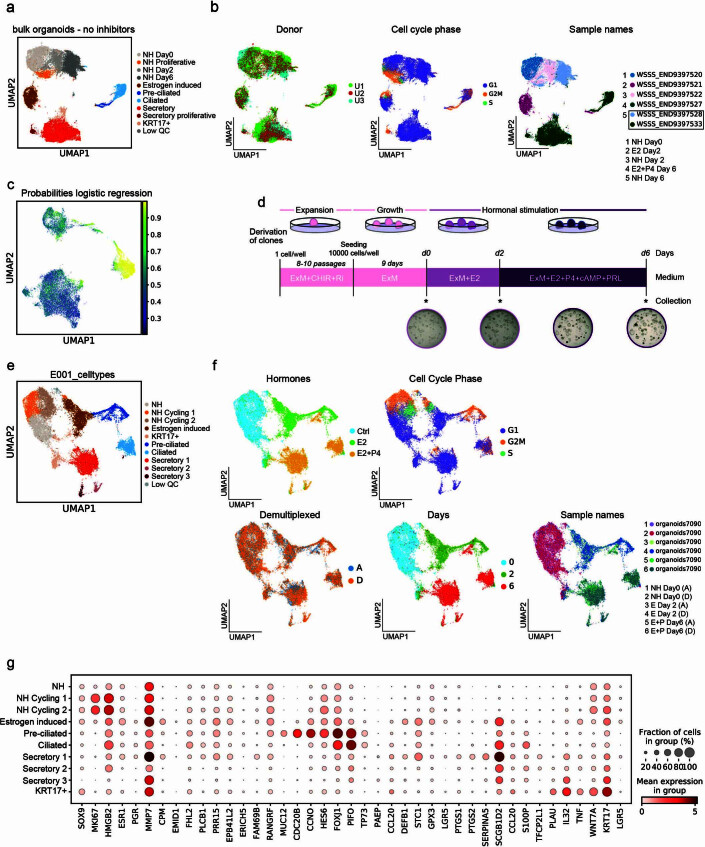
Quality control of organoid scRNA-seq dataset. **a**, UMAP (uniform manifold approximation and projection) of scRNA-seq data from all organoid samples. **b**, UMAP representations coloured by donor ID. The three donors correspond to E001, B044 and B080. **c**, Logistic regression probabilities. **d**, Experimental timeline for endometrial organoid cultures. Clonal organoids were derived in Expansion Medium (ExM) with CHIR99021 (CHIR) and ROCK inhibitor Y-22763 (Ri), grown in ExM and then subjected to hormonal stimulation with estrogen (E2) followed by E2 + progesterone (P4) + cyclic AMP (cAMP) and prolactin (PRL). The time points at which organoids were collected for scRNA-seq are shown with an asterisk. Representative bright field images of organoids for some of the timepoints are shown. **e**, UMAP projections of scRNA-seq data from two clonal organoids derived from the same individual. **f**, UMAP representations coloured by hormonal stimulation, cell cycle phase, individual clone, days after hormonal stimulation, sample ID. **g**, Dot plot showing log_2_-transformed expression of selected genes that distinguish the main cell populations. NH = No hormone; E2 = Estrogen; P4 = Progesterone; d = days.

To identify epithelial cells in the organoids, we looked for markers specific to the clusters. Before hormonal treatment, the majority of cells within the organoids are proliferative (expressing *TOP2A*, *PCNA*) and all express the estrogen receptor *ESR1* (Fig. 6b–d and Extended Data Fig. 8a,b). Two additional populations emerge when the organoids are treated with estrogen: (1) an estrogen-induced population expressing the progesterone receptor (*PGR*), a target gene of estrogen, and (2) a preciliated population sharing markers with the equivalent cluster defined in vivo. Upon further stimulation with progesterone, markers of more advanced stages of differentiation emerge in both secretory and ciliated populations. Markers of secreted products (*PAEP*, *DEFB1*) and glands (*SCGB2A2*) are both seen. Ciliated cells express typical markers (*FOXJ1*, *TP73*) and closely match their in vivo counterparts. IHC of endometrial organoids confirmed the expression of glandular and ciliary markers after hormonal stimulation (Fig. 6e).

We next assessed how closely the hormonal responses of the organoids that generate glandular and ciliated cells relate to their in vivo counterparts. To focus on the hormonal responses to estrogen and progesterone, we performed a quantitative assessment of organoids exposed to estrogen and progesterone while maintaining them in their expansion medium (ExM)^10^. Next, we projected the epithelial in vivo reference data onto the hormone-treated in vitro epithelial subsets. Assignments were made based on logistic regression predictions. In the absence of progesterone, the *SOX9*^+^ populations are the best match for estrogen-induced cells while preciliated organoid cells align with their preciliated in vivo counterparts (Fig. 6f, Extended Data Fig. 8c and Supplementary Table 9). In response to hormones, a large fraction (over a quarter of secretory cells) correspond to glandular epithelium, and all ciliated cells match perfectly with their in vivo counterparts, indicating that organoids respond similarly to hormones as in vivo (Fig. 6f and Supplementary Table 9).

To determine whether the pathways driving hormonally induced differentiation toward the ciliated and secretory lineages are similar in vitro and in vivo, we looked at the differential expression and activity of lineage-specific TFs (Fig. 6g and Supplementary Table 10). We calculated differentially expressed or active TFs in the hormonal subsets in vivo and in vitro. WNT-activated TFs (*FOXJ1*) are present in the ciliated lineage while WNT-inhibitory TFs (*CSRNP1*, *FOXO1*) are in the secretory lineage. NOTCH-induced TFs, *HEY1* and *HES1*, are activated in the secretory lineage. These results indicate that ovarian hormones activate similar pathways both in vivo and in vitro.

These results mean that it is possible to reconstruct pseudotime and recapitulate cell fate decisions of epithelial cells in response to hormones using endometrial organoids. We performed scRNA-seq on two clonal organoids from the same individual (Extended Data Fig. 8d). Both clones showed similar behavior and were integrated under the same manifold (Extended Data Fig. 8e,f). Annotation of clusters was performed based on known markers, and then clusters expressing the progesterone receptor were selected to reconstruct epithelial differentiation in response to hormones (Fig. 6h and Extended Data Fig. 8g). A subset of cells emerging from the estrogen-induced population differentiate into preciliated cells in response to estrogen and, following additional progesterone, into ciliated cells. The secretory lineage also emerges from the estrogen-induced population. Thus, there is a common progenitor for both lineages.

### WNT and NOTCH inhibition mediates epithelial differentiation

**Fig. 7:**
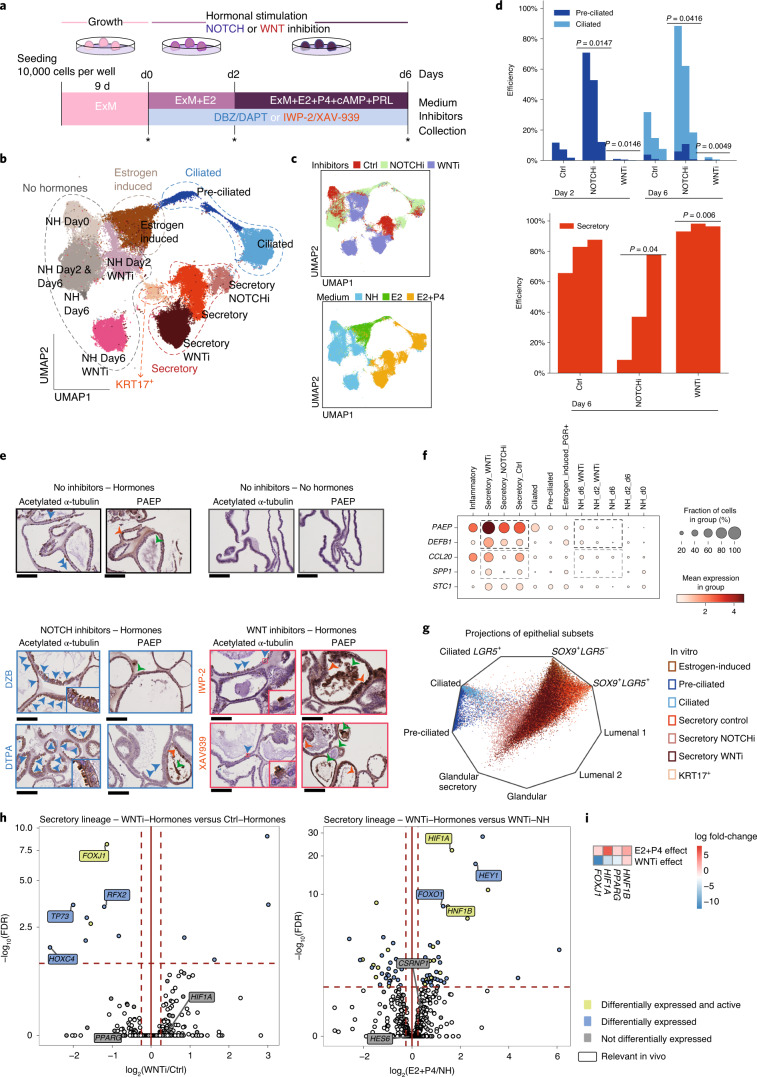
WNT and NOTCH signatures dictate endometrial epithelial differentiation. **a**, Experimental timeline of endometrial organoid cultures. Organoids were treated with inhibitors to either NOTCH (DBZ or DAPT) or WNT (IWP-2 or XAV939) upon initiation of hormonal stimulation. R-spondin-1 (RSPO-1) was omitted from ExM in the presence of WNT inhibitors. Collection time points for scRNA-seq are highlighted with asterisks. **b**, UMAP plots for scRNA-seq samples after either WNT or NOTCH inhibition. **c**, UMAP representations colored by inhibitor treatments (top) or hormonal stimulation (bottom). **d**, Bar plots showing enrichment of cells in ciliated and secretory clusters after NOTCH or WNT inhibition compared with untreated controls, analyzed with unpaired *z*-tests. **e**, IHC for acetylated α-tubulin (ciliary marker) and glycodelin (PAEP). Scale bars, 200 μm. Representative images of endometrial organoids derived from three different patients. Blue arrowheads indicate ciliated cells, orange arrowheads indicate secretory cells and green arrowheads indicate glandular secretions. **f**, Dot plot showing the log_2_-transformed expression of genes characteristic of endometrial secretions in epithelial subsets. **g**, Radial representation of the cell type probabilities predicted by a logistic model trained on epithelial cells in vivo. The linear projection shows cells in each corner whenever a cell is predicted to belong to a given class with a probability of 1. **h**, Volcano plots representing differentially expressed genes within the secretory lineage in two comparisons: (1) cells cultured with and without WNT inhibitor; progesterone is present in the media; and (2) cells cultured with and without hormones; WNT inhibitor is present in the media. TFs that are significant in the in vivo dataset are highlighted. **i**, Heatmap showing differential activities of TFs significant in the in vivo analysis. Ctrl, control; NOTCHi, NOTCH inhibitor; WNTi, WNT inhibitor.

**Extended Data Fig. 9:**
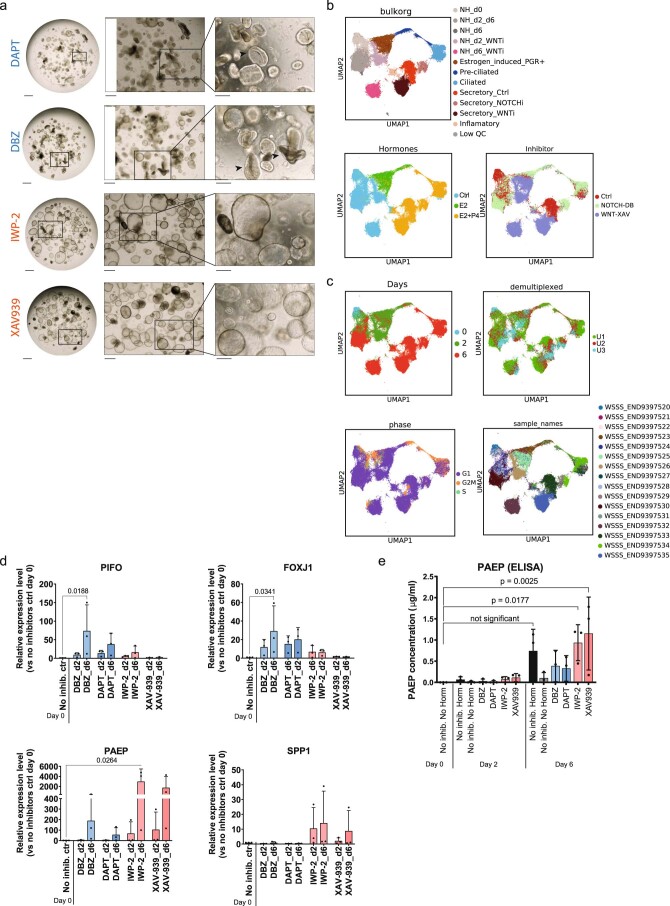
NOTCH and WNT inhibition. **a**, Representative brightfield images of the organoids treated with DBZ, DAPT, IWP-2 or XAV939 at the end of the experiment (day 6). Scale bars, 500 μm, 200 μm, 100 μm from left to right. Black arrowheads point at folded organoids. Representative images of three endometrial organoids from three different donors. **b**, UMAP (uniform manifold approximation and projection) projections of scRNA-seq data from all organoid samples. **c**, UMAP projections of scRNA-seq data coloured by days after hormonal stimulation, individual, hormonal stimulation, inhibitor used, phase of the cell cycle and sample ID. **d**, quantitative real-time PCR (qRT-PCR) analysis for genes expressed in ciliated or secretory cells of hormonally-stimulated organoids treated with NOTCH inhibitors (blue) or WNT inhibitors (pink). Bar plots showing the mean with SD levels of expression relative to housekeeping genes and control conditions at day 0 (without inhibitors, black) (n = 3 different donors). Deviations to the control conditions (without inhibitors) were detected as statistically significant by Dunnett’s multiple comparisons with p-value <0.05, which defines two-sided confidence intervals for individual deviations. **e**, ELISA assay for glycodelin (PAEP) from supernatants of hormonally stimulated organoids treated with NOTCH (blue) or WNT inhibitors (pink). Bar plots showing the mean with SD levels of expression (n = 3 donors). Deviations to the control condition (no inhibitors, no hormones) at day 0 were detected as statistically significant with Dunnett’s multiple comparisons (p-value <0.05). Source data

To test the roles of the WNT and NOTCH pathways in ciliated and secretory differentiation, we cultured organoids in the presence of inhibitors of either NOTCH (DBZ or DAPT) or WNT (IWP-2 or XAV939). We used functional, histological and single-cell transcriptomic assays to assess outcomes (Fig. 7a). Organoid viability is high under all conditions (Extended Data Fig. 9a). scRNA-seq analysis reveals a higher proportion of preciliated and ciliated cells and a lower proportion of secretory cells in the presence of DBZ (NOTCH inhibitor) (Fig. 7b–d, Extended Data Fig. 9b,c and Supplementary Table 11). This finding was validated by IHC and quantitative PCR with reverse transcription (RT–qPCR) using two NOTCH inhibitors (DBZ and DAPT) (Fig. 7e and Extended Data Fig. 9d). Ciliated cells are virtually absent when WNT is inhibited (presence of XAV939), highlighting the strong dependence on this pathway for ciliary commitment, while the proportion of secretory cells is increased under these conditions (Fig. 7b–d, Extended Data Fig. 9b,c and Supplementary Table 11). The drive toward the secretory lineage under WNT-inhibitory conditions (IWP-2 and XAV939) was further validated by RT–qPCR and IHC (Fig. 7e and Extended Data Fig. 9d). These results demonstrate the fine balance between NOTCH and WNT signaling to regulate commitment to endometrial secretory or ciliary lineages.

Hormonal stimulation in the presence of WNT and NOTCH inhibitors modified the secretory cell transcriptome (Fig. 7b). To quantify the similarity of the secretory populations that emerged in the presence of inhibitors with their in vivo counterparts, we measured the expression levels of genes encoding secretory products. Expression levels for *PAEP* and *DEFB1* are higher when WNT is inhibited and downregulated with NOTCH inhibition (Fig. 7f). We validated the increase of one secretory product, PAEP, under WNT-inhibitory conditions by ELISA (Extended Data Fig. 9e). Although *PAEP* expression levels increase slightly in the presence of WNT inhibitors even in the absence of hormones, although this is not significant (Fig. 7f). In addition, we built a logistic regression model training the data on the organoids stimulated with distinct hormonal and inhibitory conditions. Computational projection of the in vitro dataset onto the in vivo dataset shows similar overlaps of the secretory populations emerging from WNT-inhibitory conditions and controls with their in vivo counterparts (Fig. 7g and Supplementary Table 12). This shows that WNT inhibitors target a specific gene module relevant for glandular secretions.

We next dissected the regulatory programs in the secretory lineage by comparing expression and activity of TFs between populations emerging after treatment with and without hormones and WNT inhibition. The expression of NOTCH-regulated TFs (*HEY1*) is only upregulated in the presence of WNT inhibitors when hormones are present. This probably explains why WNT inhibitors are not sufficient to induce the secretory lineage on their own (Fig. 7h,i and Supplementary Table 13). In the presence of hormones, WNT inhibition represses TFs characteristic of the ciliated lineage (*FOXJ1*, *TP73*, *RFX2*) (Fig. 7h,i and Supplementary Table 13). Switching off these genes therefore drives secretory lineage differentiation.

To summarize, we show that by inhibiting NOTCH and WNT pathways it is possible to influence cell fate decisions between ciliary and secretory differentiation. This effect depends on the presence of estrogen and progesterone, and we can dissect the dialog between signaling pathways and hormonal stimulation in endometrial differentiation.

## Discussion

Profiling the uterus in space and time is essential to define the cell states and signaling pathways of normal human endometrium. This spatiotemporal reference, using samples from healthy women, will improve understanding of the molecular and cellular aberrations occurring in common conditions including infertility, endometriosis and endometrial carcinoma. The uterine lining in women of reproductive age is a challenging tissue to study due to difficulty in accessing samples covering the dynamic changes occurring across all stages of the menstrual cycle. Here, we have used single-cell expression analysis, spatial transcriptomics and high-resolution quantitative multiplex imaging to generate profiles of uterine cell states throughout the cycle. We focused on epithelial populations as they are major players in endometrial function and pathology, as suggested by our comparison of gene signatures with bulk RNA data from endometrial diseases. We use and develop computational tools to integrate and analyze scRNA-seq and spatial data and investigate the molecular mechanisms driving epithelial differentiation in the glandular and lumenal microenvironments. We utilize our reference atlas to benchmark endometrial organoids and engineer lineage-specific organoids informed by signaling factors predicted by our in vitro–in vivo comparisons. Our work shows the potential for using human cell atlases as blueprints for tissue engineering experiments.

There are two advances from our spatiotemporal uterine cell reference atlas. First, we have profiled uterine cells from transplant donors, allowing us to include the endometrial basal layer and the myometrium, which are absent from endometrial biopsies. To improve the temporal resolution of the functional endometrial layer, we have combined our dataset with another recent single-cell atlas of endometrial biopsies^22^. By integrating these two datasets, we have revealed other cell states, including a population of fibroblasts (fibroblasts C7) restricted to the basal layer. Second, our strategy of spatial mapping with 10x Genomics Visium and quantitative multiplexed smFISH techniques allows us to determine three-dimensional cellular arrangements described in transcriptomic analysis of cell isolates. By mapping cells into tissues with our deconvolution method^18^, we allocated epithelial cells into the three main endometrial layers: lumenal, functional and basal. Our expanded CellPhoneDB v.3.0 analysis framework dissects the cell signaling mechanisms in the lumenal and glandular endometrial microenvironments, which revealed that NOTCH and WNT pathways control ciliated and secretory epithelial cell commitment.

Using our single-cell transcriptomics data as a reference to deconvolute bulk data, we show that endometrial carcinomas and endometriotic lesions have a less differentiated epithelial phenotype than normal endometrium. Endometrial adenocarcinomas have two main signatures, *SOX9*^+^*LGR5*^+^ and *SOX9*^+^*LGR5*^−^, indicating that these distinct transcriptomic signatures reflect differences in pathogenesis and disease progression. *SOX9*^+^*LGR5*^+^ is the dominant signature in serous endometrial and some endometrioid adenocarcinomas and is positively associated with the ‘Copy-Number high’ molecular subtype from TCGA, as well as the clinically more aggressive stage III and IV adenocarcinomas. Our study is consistent with previous analyses showing that serous adenocarcinomas share molecular features with a subset of endometrioid tumors^30^. We also show an enrichment of markers from *SOX9*^+^*LGR5*^+^ epithelial cells in endometriotic lesions, in line with findings that organoids derived from higher stages (III–IV) of endometriosis have higher expression of *SOX9* (ref. ^35^). These organoid models will allow investigation of the potential roles of these *SOX9* populations in driving endometrial cancer, endometriosis and other disorders^35^.

Endometrial organoids are a powerful model to study normal endometrial epithelium^10,11,36^. We have systematically benchmarked the cellular composition of organoids relative to our in vivo reference map. Machine learning approaches, such as logistic regression scoring of expression profiles as well as correlation analysis, have been previously used to compare in vitro datasets with their in vivo counterparts^37,38^. Using a logistic regression approach, we demonstrate that endometrial organoids recapitulate the in vivo response to hormones, with the ciliary lineage becoming fully differentiated while the secretory lineage maintains progenitors in addition to differentiated secretory cells. This provides compelling evidence for the validity of our model. The organoids continue to expand during exposure to ovarian hormones because they are cultured in ExM that promotes proliferation. We also compared TFs operating in vivo and in vitro and showed that similar programs are induced. Informed by these findings, further optimization of the culture conditions to achieve more complete secretory differentiation is underway. Our computational kit for in vivo and in vitro comparisons will be of general utility for tissue engineering experiments using Human Cell Atlas data as a blueprint.

Our extensive validation assessing the hormonal responses of the endometrial organoids means that we could use them to test the effects of NOTCH and WNT signaling on epithelial cell fate^10^. Inhibition of WNT signaling, which mimics the low-WNT microenvironment in differentiated glands, inhibits ciliary commitment and induces secretory cells. In the presence of hormones, these cells produce more secretions, probably through stronger silencing of ciliary genes. WNT inhibition alone does not result in secretory differentiation, as the NOTCH pathway is not induced without hormonal stimulation. These results reinforce previous findings suggesting tight coordination between these signaling pathways and ovarian hormones^39,40^. NOTCH inhibitors were found to promote the generation of ciliated cells in fallopian tube^41^ and endometrial organoids^42,43^. We also show that NOTCH-inhibited secretory cells express lower amounts of uterine milk proteins. Using single-cell mapping, we pinpoint the effect of NOTCH to early ciliary differentiation, as suggested by the strong effect NOTCH inhibition has on preciliated cells. Thus, we demonstrate opposing roles of WNT and NOTCH in shaping distinct endometrial epithelial lineages. In vivo, this is regulated by the boundaries set by the localization of distinct cellular populations in the lumenal versus glandular microenvironments.

Our integrative map of cellular profiles of the normal endometrium will serve as an essential reference for the study of many neglected endometrial disorders. Organoids, which can be biobanked, have been established from samples of endometriosis and endometrial adenocarcinomas that resemble the original tumors^10,11,35,44^. Our study shows that the combination of genomics, imaging and organoids can create a robust platform for studying endometrial physiology. This will have a wide-ranging impact on women’s health and reproductive medicine.

## Methods

### Uterine tissue retrieval

Full-thickness uterine wall samples were obtained from deceased transplant organ donors (A13, A30) after ethical approval (reference 15/EE/0152, East of England–Cambridge South Research Ethics Committee) and informed consent from the donor families. Uterus was removed within 1 h of circulatory arrest.

Full-thickness uterine wall samples were collected from four women during autopsy (Trv2, Trv3, Trv4 and Trv5). All four individuals died of noncancer-related causes, either traumatic injuries (Trv2, Trv3 and Trv4) or brain edema (Trv5). Samples were collected within less than 10 h of death (postmortem interval was 5, 2, 4 and 6 h, respectively). Once collected, all tissue biopsies were snap-frozen in liquid nitrogen and subsequently stored at −80 °C. The use of these tissues was approved by the London, Surrey Research Ethics Committee (REC reference 17/LO/1801, 26/10/2017).

Endometrial biopsies were obtained from live donors with written, informed consent from all participants from multiple centers.

Endometrial biopsies for sequencing were obtained from individuals recruited from Newcastle Upon Tyne Hospitals after ethical approval (reference 16/NE/0167, North East–Newcastle & North Tyneside 1 Research Ethics Committee).

Proliferative endometrial biopsies for deriving organoids were obtained from Addenbrooke’s Hospital under ethical approval from the East of England–Cambridge South Research Ethics Committee (08/H0305/40).

Endometrial scratch samples from secretory-phase endometrium for deriving organoids were obtained from Bourne Hall Clinic under ethical approval from the East of England–Central Research Ethics Committee for the ‘Biology of the Human Uterus in Pregnancy and Disease Tissue Bank’ run by the Centre for Trophoblast Research (17/EE/0151).

Endometrial biopsies were obtained using a disposable endometrial cell sampler, starting from the uterine fundus and moving downward to the internal cervical ostium. None of the participants were on hormonal treatments for at least 3 months before the procedure.

Endometrial tissues were staged based on standard histological criteria.

Tissue dissociation for all fresh tissues was conducted within 24 h of tissue retrieval in a two-step digestion protocol. Briefly, endometrial tissue was treated with collagenase to retrieve the stromal fraction (step 1)^45^. After filtering, pieces of tissue retained on the 100-µm filter were treated with trypsin to enrich for the epithelial fraction (step 2)^46^. Nuclei were released via Dounce homogenization as described previously^47^.

### Endometrial organoid cultures

Endometrial organoids were grown as previously described^10^. Briefly, organoids were cultured at 37 °C in a humidified atmosphere of 5% CO_2_. The medium was refreshed every 2–3 d, and the organoids were passaged at an average ratio of 1:3 every 5–7 d. The organoid suspension was centrifuged for 6 min at 600*g* between passaging steps. The passaged organoid pellet was resuspended in 25-μl ice-cold Matrigel (Corning, 356231) droplets, plated in a 48-well plate (Costar, 3548), allowed to solidify at 37 °C for 15–30 min and covered with 250 μl of endometrial organoid ExM. Components of ExM for culturing human endometrial organoids are available in Supplementary Table 14.

### Hormonal stimulation and inhibition experiment of endometrial organoids

Endometrial organoids were stimulated with hormones and treated with NOTCH γ-secretase inhibitors (DBZ, Tocris 4489 and DAPT, Tocris 2634) as well as WNT inhibitors (tankyrase inhibitor XAV939, Tocris 3748 and porcupine inhibitor IWP-2, Tocris 3533) for 6 d. First, 10,000 single cells were plated per 25-μl Matrigel droplet into a 48-well plate with ExM supplemented with Rho kinase inhibitor (Y-27632-CAS 146986-50-7) and CHIR 99021. At 10 d after plating, organoids were primed with 10 nM estrogen (E2) and treated with NOTCH and WNT inhibitors (20 μM DAPT, 1 μM DBZ, 2 μM IWP-2, 2 μM XAV939 in the ExM). R-spondin-1, a WNT signaling activator, was depleted from the ExM in the conditions where WNT inhibitors were used. After 48 h, they were stimulated with 10 nM E2, 1 μM progesterone (P4), 100 μg ml^−1^ cAMP and 20 ng ml^−1^ prolactin while still being treated with NOTCH and WNT inhibitors (20 μM DAPT, 1 μM DBZ, 2 μM IWP-2, 2 μM XAV939). Conditions in which (1) inhibitors but no hormones, (2) no inhibitors but hormones and (3) no inhibitors and no hormones were added were used as controls.

### Doublet detection, alignment of data across different batches and clustering of scRNA-seq data

The 10x Genomics scRNA-seq data were analyzed with Scanpy^48^, with the pipeline following their recommended standard practices. In addition, we implemented a number of enhancements described below.

Individual samples of single cells or single nuclei were initially analyzed separately before being batch corrected into an integrated dataset and had two-step diffusion doublet identification performed^49,50^. The first step was performed with Scrublet^51^ (v.0.2.1) on a per-sample basis, with the scores diffused by overclustering the cells and reporting each cluster’s median value. Doublets were identified from a distribution of these scores centered at the median and using a mean absolute standard deviation estimate, with statistically significant cells after FDR correction flagged as doublets. The second diffusion step takes place in a joint multi-sample manifold, with the frequency of identified doublets in granular (Leiden resolution 10) clusters serving as the basis for the distribution and the statistical significance analysis described in ref. ^50^, with Bonferroni for FDR correction and a significance threshold of 0.01. In the organoid samples, additional cells were identified as doublets by Souporcell^52^ when multiple genotypes were found in single droplets.

After filtering out cells with fewer than 500 genes and more than 15% mitochondrial reads (20% for organoids), samples were integrated using scVI^53^ (v.0.6.5). While the raw count matrices were used for single cells, the counts from single nuclei were denoised from ambient RNA before the manifold identification. For that task, decontX^54^ (under the ‘celda’ R package v.1.5.11) was used on each sample separately. We then excluded cell cycling genes from G2/M and S phases listed inside the Seurat package^55^. For the in vivo dataset, the expression levels of the 5,000 genes that were identified by the scVI native method were modeled by its generative model with 64 latent variables for 500 iterations. Epithelial, endothelial and immune cells were subsequently reanalyzed separately by using 16 latent variables. The resulting latent variables were used for neighbor identification for Leiden clustering^56^ and uniform manifold approximation and projection (UMAP) visualization. The resulting clusters specific to a single donor, or that had lower numbers of genes expressed or a lower percentage of mitochondrial expression, were excluded. In the epithelial and immune cell reanalysis, cells exhibiting high doublet scores were excluded, and epithelial cells were subsampled to balance donor contribution (Annotation of organoid data using scRNA-seq in vivo dataset reference).

### Annotation of scRNA-seq datasets

Identification and labeling of the major cell types in the in vivo dataset was by manual inspection of marker genes and interpretation of these based on the literature. Cluster-specific marker genes were defined using two approaches. To account for the donor effect, we first used DEseq2 (ref. ^57^), where cells were aggregated into in silico mini-bulks by summing the raw expression of single cells separately according to their donor origin. Every cell-type-specific mini-bulk was compared against a matched mini-bulk that corresponded to all other cells from the same donor; however, such mini-bulk pairs defined by aggregating less than ten cells were excluded (for example, clusters associated with a specific menstrual stage). Secondly, the Wilcoxon test was used to report genes that were differentially expressed. To account for change of sequencing depth, cells were partitioned into four groups corresponding to the quartiles of the sequencing depth of cells considered (independent of donors), and the four resulting Z-scores were combined. *P* values were adjusted with the Benjamini–Hochberg method.

For each cell, we estimated the cell cycle phase (G1, S or G2/M) based on its expression of G2/M and S phase markers, following the method described in ref. ^58^ and implemented in Scanpy score_genes_cell_cycle function.

### Efficiency of organoid differentiation

To identify clusters predominantly appearing in organoid cultures upon treatment with WNT or NOTCH inhibitors, we evaluated the proportion of cells in each cluster coming from the organoids with and without inhibitors using Fisher’s exact test. Odds ratios were computed against the control organoids (grown with no inhibitors) at the matched time points on a per-genotype basis. In addition, the robustness of the observed effect was evaluated using a paired *t*-test that compares these fractions of cells from organoids that were treated with inhibitor with their respective controls.

### Annotation of organoid data using scRNA-seq in vivo dataset reference

To identify transcriptomic similarities with the in vivo scRNA-seq epithelial subset, we used a regularized logistic regression approach. To best link variation in gene expression in organoids to changes in donors with known stages of the menstrual cycle, we subsampled the in vivo epithelial cells up to 1,000 cells per donor, which balances the cell number representing each stage. To limit the influence of cell cycle, we excluded the SOX9 proliferative cluster composed of a majority of cells at G2/M or S phase, and also excluded the G2/M and S genes from Seurat. Subsequently, we subset both datasets to their shared highly variable genes and further prune half of remaining genes that are not cell-type-specific according to three heuristic measures (fold-increase, fold-increase × fraction-positive, fold-increase × fraction-positive^0.5^)^59^. Gene expression was log-transformed and normalized by the maximum RNA expression for both datasets. The model was trained with the in vivo epithelial identities, with 10,000 iterations, and used to classify organoid cells. To visualize results as radial projection we followed La Manno et al.^37^, where the overlaid position of each cell corresponds to the weighted average of radially balanced unitary vectors (each pointing toward a different corner of a regular polygon), where the weight is each posterior probability of a cell to belong to a given cell type. The latter transformation is a softmax function where each component is multiplied by 15 before being exponentiated.

### Annotation of snRNA-seq using scRNA-seq in vivo dataset as a reference

A quantitative measure of the similarity was reported by evaluating the cosine distance from in vivo single nuclei to the centroids of expression defined by the cell-type clusters identified in in vivo single cells, where each vector compared either contains expression of all selected genes in a single cell or their respective mean expression for a given cell-type cluster. Results were visualized with a radial projection as described previously by La Manno et al.^37^.

### Trajectory analysis of organoid data

To identify the branching point where cells commit to a ciliated or secretory fate, we first identified cell clusters in the organoid cultures. Then, we used Palantir^60^ (v.1.0.0) on cells from the clusters that did exhibit a mean expression level of the progesterone receptor higher than 0.2. A randomly selected cell corresponding to proliferating cells at day 2 was selected as a cell of origin.

### Cellular signal analysis

Tumor bulk transcriptomes for endometrioid (430 samples) and serous (112 samples) endometrial adenocarcinoma were downloaded from TCGA. Cellular signal analysis was then applied^61^ to identify the major transcriptional programs used by tumor cells based on our single-cell endometrial atlas. This method fits the raw bulk messenger RNA counts to a weighted linear combination of transcriptomic signals derived from reference single-cell data. To limit the effect of the cell cycle, we only included cells in the G1 phase and excluded a proliferative *SOX9* cluster. Proportions of epithelial-derived signals in the bulk samples were computed as the fraction of samples for which signals (exposures) derived from an epithelial cell cluster exceed the intercept term of the model. Clinical data associated with the samples that exhibited exposure for *SOX9*^+^*LGR5*^+^ or *SOX9*^+^*LGR5*^−^ above the intercept value were further investigated; we used the Kruskal–Wallis test to confirm that *SOX9*^+^*LGR5*^+^ signature contribution (exposure) in tumors differs in different cancer stages (as defined in TCGA clinical data), and used the Wilcoxon test and *t*-test to assess the significance of the increase in exposure noted in later stages of cancer, as well as several clinical data (Supplementary Table 3).

### Location of cell types in Visium data

To spatially map cell types defined by scRNA-seq analysis within the Visium spatial transcriptomics data, we used cell2location (ref. ^18^) (v.0.5-alpha). Briefly, cell2location decomposes multi-cell spatial transcriptomics data into cell-type abundance estimates in a spatially resolved manner. First, the model derives expression signatures of cell types by calculating average expression counts of each gene in each cell type in the raw count scRNA-seq data, selecting genes expressed in at least three cells. Next, to obtain cell-type locations, the model performs a hierarchical non-negative decomposition of the gene expression profiles at spatial locations (spots with multiple cells) into the reference signatures. Each Visium section was analyzed separately with parameters set to default values, except train_args = ‘n_iter’: 30,000; posterior_args = ‘n_samples’: 1,000; model_kwargs = ‘cell_number_prior’: {‘cells_per_spot’: 8, ‘factors_per_spot’: 4}; and ‘gene_level_prior’: {‘mean’: 1/2, ‘sd’: 1/4, ‘mean_var_ratio’: 1}. We visualize the absolute amount of mRNA contributed by each cell population to each spot. We used a 5% percentile of the posterior distribution of this parameter (mRNA counts), representing the number of mRNA molecules confidently assigned to each cell type.

### Clustering of spots

Visium data were processed using Scanpy^48^ following the recommended tutorial with normalization using a scaling factor of 10,000; log transformation; variable gene detection with ‘Seurat’ flavor; principal-component analysis (PCA); neighborhood graph building; and UMAP calculation. Each sample was analyzed independently.

Clusters were defined by the Louvain algorithm and assigned as myometrium or endometrium based on visual inspection of the H&E image of the tissue aligned with each spot. The cluster of spots corresponding to epithelial cells in the endometrium for sample A30, 152807 slide, was further clustered using the same approach. One of the spot subpopulations was excluded due to the low percentage of epithelial cells in the spot after visual inspection. The other ‘epithelial’ spot subpopulations were labeled based on the endometrial layer in which they were found—basal, lumenal and glandular.

Differentially expressed genes in each subpopulation of ‘epithelial’ spots were calculated using the limma R package^62^.

### Calculating TF activities

TF activities were estimated via the combined expression levels of their targets. Target genes were retrieved from Dorothea^32^, where TF–target relationships are scored from A to E, with decreasing confidence. Here we updated Dorothea regulons as follows: first, synonymous gene names were corrected; second, bona fide TF–target relationships manually curated from Uniprot were added as a new curated source; third, signed and curated interactions were upgraded to score B; fourth, TRRUST^63^ curated interactions were updated to v.2_20180416 version and signed interactions supported by more than one PubMed source were upgraded to A; and, finally, we created a new category (AA) for the most trustable TF–target interactions that were either detected by all approaches or in more than two curated resources. For each TF, we used the highest scored set of targets with at least ten target genes, as in the original publication^32^.

Next, we estimated TF activities by performing a Gene Set Enrichment Analysis (GSEA)-like analysis of the gene expression signatures of each cluster resulting from the Wilcoxon test (Annotation of scRNA-seq datasets) with the *msVIPER* function in the Viper R package^64^ v.1.22.0. New regulons are available in Supplementary Table 15.

### CellPhoneDB v.3.0

To study the interactions between epithelial and other cell populations identified in our endometrial samples, we updated our CellPhoneDB approach to v.3.0 (ref. ^33^). First, we retrieved the interacting pairs of ligands and receptors satisfying the following criteria: (1) all the members were expressed in at least 10% of the cells in the cluster under consideration and (2) at least one of the members in the ligand or the receptor is a differentially expressed gene (Wilcoxon test; Annotation of scRNA-seq datasets). To account for the distinct temporal and spatial location of cells (that is, microenvironment), we further classified the epithelial interactions based on (1) the phase of the menstrual cycle where cell subsets coexist and (2) their location in the three main endometrial layers (luminal, glandular and basal) according to cell2location (ref. ^18^).

To account for the complexity of WNT cell–cell signaling, several ligands and functional heteromeric receptors were further curated manually and re-annotated in the CellPhoneDB database (Supplementary Table 16).

### Image stitching and manual annotation of selected glands

Confocal image stacks were stitched as two-dimensional maximum intensity projections using the BIOP Perkin Elmer Acapella Stitcher (EPFL, Lausanne; https://www.perkinelmer.com/PDFs/downloads/TCH-Workflows-In-Depth-High-Content-Analysis-Operetta.pdf).

### smFISH quantification

RNA spot quantification of smFISH targeting WNT7A and NOTCH2 was achieved with a three-step process:

Step 1. Segmenting glands within the tissue. Ilastik^65^ was used to train a random-forest-based pixel classifier to detect valid gland areas based on the Nuclear-DAPI channel and the Gland-EPCAM (IHC) channel. Three rounds of ilastik classification were used to achieve adequate rejection of off-target signal to segment only the glands.

Step 2. Segmenting RNA spots within the glands. Another ilastik pixel classifier was used on the spot channels to segment areas that corresponded to genuine spots that were situated in the glands segmented in step 1. The spots were verified by only including spots visible in one channel only. This was done to remove blood inclusions, which gave a confounding signal across multiple channels.

Step 3. The edge of the lumen was manually annotated using napari^66^. The distance of each pixel on the image was calculated to the nearest point on the lumen edge. Then the total fluorescence intensity was measured for spots in glands and binned into intervals of distance away from the lumen. The gland area was also calculated for each distance interval. The spot fluorescence was divided by the gland interval to give a value of spot intensity that was normalized by area.

### Reporting Summary

Further information on research design is available in the Nature Research Reporting Summary linked to this article.

## Online content

Any methods, additional references, Nature Research reporting summaries, source data, extended data, supplementary information, acknowledgements, peer review information; details of author contributions and competing interests; and statements of data and code availability are available at 10.1038/s41588-021-00972-2.

## Supplementary information

Supplementary Information Supplementary Methods, statistics and reproducibility, and supplementary references.

Reporting Summary

Supplementary Tables. Table 1: Metadata of samples, including biopsies and deceased transplant donors, for scRNA-seq, snRNA-seq and Visium analysis. Table 2: Excel file containing the quality control uterine atlas. Table 3a: Relative contribution of single-cell-derived signals from healthy endometrium in explaining bulk transcriptomes of endometrial cancers (430 endometrioid and 122 serous endometrial). Table 3b: Correlations of SOX9^+^LRG5^+^ exposures to clinical TCGA data found within the 313 endometrial adenocarcinomas that have an exposure for either SOX9^+^LRG5^+^ or SOX9^+^LRG5^−^ above the intercept value. Statistical significance of these correlations is reported by a two-sided *t*-test and by a two-sided Wilcoxon test. Table 3c: Table containing metadata of patients with endometriosis from GSE141549 included in our analysis. Table 4a: Table containing differentially expressed genes in the main epithelial subpopulations detected in the scRNA-seq dataset. Statistical significance of differentially expressed genes is reported with DESeq2 and Wilcoxon, where reported *P* values correspond to two-sided tests before and after FDR correction. Table 4b: Table containing Dorothea TF activities estimated in the epithelial subpopulations in the scRNA-seq dataset. Each row represents a TF/cluster analysis. Table 4c: TFs predicted to be both differentially active and expressed. Table 5: Table containing differentially expressed genes in the main epithelial clusters detected in the Visium dataset. Reported *P* values correspond to two-sided tests before and after FDR correction. Table 6a**:** Differentially expressed genes in the main and epithelial subsets in vivo. Statistical significance of differentially expressed genes is reported with DESeq2 and Wilcoxon, where reported *P* values correspond to two-sided tests before and after FDR correction. Table 6b–f: Table containing CellPhoneDB interactions (epithelial–epithelial & fibroblasts–epithelial) predicted for the lumenal proliferative, lumenal secretory, functional proliferative, functional secretory and basal microenvironments. Columns represent interaction cell pairs, and rows represent the interactions. Table is binary, with 1 indicating that all the members of the interaction are expressed in at least 10% of cells and at least one member is a differentially expressed gene in any cell type in the pair with a positive log fold change and FDR < 0.001. Table 7: Metadata of samples to derive endometrial organoids. Table 8: Quality control details of the scRNA-seq dataset obtained from organoid cultures, which detail the list of samples obtained with hormone and inhibitor treatments. Table 9a: Number of cells from the organoid populations predicted to correspond to cell type classes learned by a logistic regression model defined using the in vivo epithelial cells as a reference. Table 9b: Corresponding confusion matrix for the proportion of cells predicted in each class. Table 9c: Expected in vivo epithelial class posterior probability for a cell randomly sampled from a cell type cluster defined in the organoid cultures. Table 10a: Table containing differentially expressed genes in the organoid experiment detected by scRNA-seq. Statistical significance of differentially expressed genes is reported with DESeq2 and Wilcoxon, where reported *P* values correspond to two-sided tests before and after FDR correction. Table 10b: Table containing Dorothea TF activities estimated for the organoid subpopulations. Table 10c,d: Transcription factor comparison between epithelial subpopulations in vivo and organoid experiment for the glandular and ciliated lineages, respectively. Table 11a,b: Table containing the cell counts and corresponding proportions identified as estrogen-induced, preciliated, ciliated or secretory in the organoids on days 2 and 6, in relation to the three genotypes and inhibitor treatments considered targeting NOTCH and WNT. Table 11c: Table containing the fold change for the frequency of a given cell type observed from the influence of an inhibitor treatment. Table 11d,e: The statistical significance of detected changes was reported for all three genotypes independently using the hypergeometric test, and robustness of the detected effects across genotypes was then evaluated using a paired *t*-test for the three efficiencies reported without inhibitor treatment against the three reported with NOTCH and WNT inhibitors (one-sided tests). Table 12a: Number of cells from the organoid populations, which include organoids treated with NOTCH and WNT inhibitors, predicted to correspond to cell type classes learned by a logistic regression model defined using the in vivo epithelial cells as a reference. Table 12b: Corresponding confusion matrix for the proportion of cells predicted in each class. Table 12c: Expected in vivo epithelial class posterior probability for a cell randomly sampled from a cell type cluster defined in the organoid cultures. Table 13a: Table containing two pairwise differential expression analyses in the organoid experiment with inhibitors: (1) measuring E + P effect in the secretory WNTi population where the *Secretory_WNTi* population is compared against *NH_WNTi* and (2) measuring WNTi effect in the secretory lineage where the *Secretory_WNTi* population is compared against *Secretory_Ctrl*. Statistical significance of differentially expressed genes is reported with DESeq2 and Wilcoxon, where reported *P* values correspond to two-sided tests before and after FDR correction. Table 13b: Table containing Dorothea TF activities estimated from DEGs resulting from the Wilcoxon test reported in sheet (13a). Table 14: Components of ExM for culturing human endometrial organoids. Table 15a: New manually curated TF–target interactions. Table 15b: Table containing TF regulons and scores used to quantify TF activities from differentially expressed genes. Table 16: Table containing new curated CellPhoneDB interactions. Table 17: TaqMan probes used for RT–qPCR. Table 18a: List of primary antibodies. Table 18b: List of secondary antibodies. Table 19: Probes used for smFISH.

## Source data

Source Data Fig. 4 Statistical source data.

Source Data Extended Data Fig. 9 Statistical source data.

## Data Availability

Datasets were uploaded into ArrayExpress under accession numbers E-MTAB-10287 (scRNA-seq in vivo), E-MTAB-9260 (Visium in vivo) and E-MTAB-10283 (scRNA-seq in vitro). snRNA-seq in vivo data were uploaded into the European Genome-phenome Archive; EGAD00001007909. Requests for data access should be sent directly to the Data Access Committee of this work: datasharing@sanger.ac.uk (https://ega-archive.org/datasets/EGAD00001007909). Tumor bulk transcriptomes for endometrioid and serous endometrial adenocarcinomas were downloaded from TCGA. Additional single-cell transcriptomes of ten endometrial biopsies were downloaded from the Gene Expression Omnibus with GSE111976 (ref. ^22^). Processed matrices can be accessed and downloaded from www.reproductivecellatlas.org. Image datasets are available at the EMBL-EBI BioImage Archive under accession number S-BIAD190. Data availability includes all datasets in the manuscript. Source data are provided with this paper.

## Code availability

Code is available at https://github.com/Ventolab/UHCA. Source code and tutorials for CellPhoneDB v.3 are available at https://github.com/Ventolab/CellphoneDB.
