# Author Correction: Mapping the temporal and spatial dynamics of the human endometrium in vivo and in vitro

**DOI:** 10.1038/s41588-022-01287-6

**Published:** 2022-12-20

**Authors:** Luz Garcia-Alonso, Louis-François Handfield, Kenny Roberts, Konstantina Nikolakopoulou, Ridma C. Fernando, Lucy Gardner, Benjamin Woodhams, Anna Arutyunyan, Krzysztof Polanski, Regina Hoo, Carmen Sancho-Serra, Tong Li, Kwasi Kwakwa, Elizabeth Tuck, Valentina Lorenzi, Hassan Massalha, Martin Prete, Vitalii Kleshchevnikov, Aleksandra Tarkowska, Tarryn Porter, Cecilia Icoresi Mazzeo, Stijn van Dongen, Monika Dabrowska, Vasyl Vaskivskyi, Krishnaa T. Mahbubani, Jong-eun Park, Mercedes Jimenez-Linan, Lia Campos, Vladimir Yu. Kiselev, Cecilia Lindskog, Paul Ayuk, Elena Prigmore, Michael R. Stratton, Kourosh Saeb-Parsy, Ashley Moffett, Luiza Moore, Omer A. Bayraktar, Sarah A. Teichmann, Margherita Y. Turco, Roser Vento-Tormo

**Affiliations:** 1 Wellcome Sanger Institute, Cambridge, UK; 2 Centre for Trophoblast Research, University of Cambridge, Cambridge, UK; 3 Department of Pathology, University of Cambridge, Cambridge, UK; 4 EMBL-EBI, Wellcome Genome Campus, Hinxton, UK; 5 Theory of Condensed Matter Group, Cavendish Laboratory, University of Cambridge, Cambridge, UK; 6 Department of Haematology, University of Cambridge, Cambridge, UK; 7 Cambridge Biorepository for Translational Medicine (CBTM), NIHR Cambridge Biomedical Research Centre, Cambridge, UK; 8 Department of Pathology, Cambridge University Hospitals NHS Foundation Trust, Cambridge, UK; 9 Department of Immunology, Genetics and Pathology and Science for Life Laboratory, Uppsala University, Uppsala, Sweden; 10 Department of Women’s Services, Newcastle-upon-Tyne Hospitals NHS Foundation Trust, Newcastle upon Tyne, UK; 11 Department of Surgery, University of Cambridge, Cambridge, UK; 12 Present Address: Friedrich Miescher Institute for Biomedical Research, Basel, Switzerland

**Keywords:** Cell biology, Biochemistry

Correction to: *Nature Genetics* 10.1038/s41588-021-00972-2, published online 2 December 2021.

In the version of this article initially published, “European Research Council grant no. 853546” was missing from the Acknowledgements section. The error has been corrected in the HTML and PDF versions of the article.

