# Supplementary material for: Mapping the temporal and spatial dynamics of the human endometrium in vivo and in vitro
Source: Nat Genet. 2021 Dec 2;53(12):1698–711. doi: 10.1038/s41588-021-00972-2 (PMC8648563; doi:10.1038/s41588-021-00972-2)
Supplement: Supplementary file 1 — Supplementary Methods, statistics and reproducibility, and supplementary references. [file 41588_2021_972_MOESM1_ESM.pdf]

nature portfolio

https://doi.org/10.1038/s41588-021-00972-2

Supplementary information

Mapping the temporal and spatial dynamics of the human endometrium in vivo and in vitro

In the format provided by the authors and unedited

Supplementary Note

Supplementary Methods

Tissue processing

All tissues for sequencing and spatial work were collected in saline (HypoThermosol biopreservation media) and stored at 4°C until processing. Tissue dissociation for all tissues was conducted within 24 hours of tissue retrieval in a two-step digestion protocol.

Step-1 Collagenase treatment[45]: Tissue was transferred to a sterile 10 mm^2 tissue culture dish and cut into <1 mm^3 segments before being transferred to a 50 ml conical tube. Tissues were digested with 1 mg/ml collagenase type V in RPMI (Sigma-Aldrich), 0.1 mg/ml DNaseI (Sigma-Aldrich) supplemented with 10% (v/v) heat-inactivated fetal bovine serum (FBS; Gibco), 100 U/mL penicillin (Sigma-Aldrich) for 45 minutes at 37°C with intermittent shaking. Digested tissue was passed through a 100 µm filter, and cells collected by centrifugation (450×g for 5 minutes at 4°C). Cells were treated with 1X red blood cell (RBC) lysis buffer (eBioscience) for 5 minutes at room temperature and washed with flow buffer (PBS containing 5% (v/v) FBS and 2 mM EDTA) prior to cell counting. Cells were loaded into a 10x reaction (stromal enriched fraction).

Step-2 Trypsin treatment[46]: Pieces of tissue retained on the 100 µm filter were washed with PBS and further digested with Trypsin/EDTA 0.25% for 20 minutes at 37°C with intermittent shaking. Digested tissue was passed through a 100 µm filter, and cells collected by centrifugation (450×g for 5 minutes at 4°C). Cells were washed with flow buffer (PBS containing 5% (v/v) FBS and 2 mM EDTA) prior to cell counting. Cells were loaded into a 10x reaction (epithelial enriched fraction).

Tissue freezing

Fresh tissue samples were embedded in cold OCT medium and flash frozen using a dry ice-isopentane slurry[67].

Nuclei extraction

Thick (200 µm) uterine sections were cryosectioned, dissected from OCT and kept in a tube on dry ice until subsequent processing. Nuclei were released *via* Dounce homogenisation as described previously[47].

Endometrial organoid dissociation

Matrigel was removed using Cell Recovery Solution (Corning, 354253) for 1 hour on ice. Organoids were washed with cold PBS and broken up by 300 strokes of an automatic pipette (Eppendorf, Xplorer Plus 613-223), then incubated, first with pre-warmed accutase cell detachment solution (Corning, 25-058-CI) for 5 minutes at 37°C, and second with collagenase V (Sigma-Aldrich, C-9263) diluted in 10% FBS/Advanced DMEM/F12 for 15 minutes at 37°C. The digest was passed through a 40 μm nylon mesh cell strainer to purify a single-cell suspension. Where undigested fragments were present, the collagenase step was repeated. The final digest suspension was resuspended in Expansion Medium (ExM). Cells were diluted in trypan blue for live and dead cell counting using a haemocytometer.

RNA extraction, cDNA synthesis and qRT-CR

Total RNA was extracted using the RNeasy Micro Kit with on-column DNase treatment (Qiagen, 76004), following manufacturer's instructions. RNA was resuspended in 14 μl RNase-free water, and purity and concentration were determined using a NanoDrop 1000 spectrophotometer (Thermo Fisher Scientific). For cDNA synthesis, 500-1000 ng of total RNA was reverse transcribed using SuperScript VILO cDNA Synthesis Kit (Thermo Fisher Scientific, 11754050) following manufacturer's instructions. The extracted RNA was diluted into 5X VILO Reaction Mix containing random primers, dNTPs, and $MgCl_2$ and 10X SuperScript III Enzyme Blend containing SuperScript III Reverse Transcriptase, RNaseOUT Recombinant Ribonuclease Inhibitor, and proprietary helper protein. Reactions were then incubated for 10 minutes at 25°C, 1 hour at 42°C and 5 minutes at 85°C. A no-reverse transcriptase reaction was prepared for use as a control for genomic DNA contamination. Quantitative real-time PCR (qRT-PCR) was performed on a 7500HT Fast Real-Time PCR system (Applied Biosystems) using TaqMan Fast Advanced Master Mix (Thermo Fisher Scientific, 4444557) and Taqman gene-specific primer probes, following manufacturer's protocol. Initial denaturation was performed for 20 seconds at 95°C, followed by 40 amplification cycles of 3 seconds at 95°C and 30 seconds at 60°C. Each qRT-PCR reaction was performed in duplicate, alongside non-template controls. Expression levels were calculated using the comparative cycle threshold (Ct) method. Normalised expression levels were calculated as $2^{-\Delta Ct}$ where ΔCt = Ct (gene of interest) – Ct (geometric mean of housekeeping genes *HPRT1*, *TOP1*, and *TBP*). Statistical analyses were performed using GraphPad Prism 5.03 software. All data are represented as mean ± standard deviation (SD). Statistical difference between compared groups was evaluated by ordinary unpaired one-way ANOVA for multiple groups compared to control treatments.

Taqman probes used for RT-qPCR available at **Supplementary Table 17.**

Immunohistochemistry

Organoids were separated from Matrigel using the Cell Recovery Solution (Corning, 354253), washed with cold PBS, fixed in formalin (Sigma, F5554), embedded into 2% agarose (Melford, MB1200) and finally embedded into paraffin and sectioned at 4 μm thickness. The tissue and organoid sections were dewaxed with Histoclear (National Diagnostics, HS-200), cleared with 100% ethanol and rehydrated serially through 90%, 70%, 50% ethanol to PBS. Epitope retrieval was conducted in Access Revelation (AR) pH 6.4 (A.Menarini, MP-607-PG1) citrate buffer or Access Super (AS) pH 9 (A.Menarini, MP-606-PG1) Tris-EDTA buffer at 125°C in an Antigen Access pressure cooker (A.Menarini, MP-2008-CE). Sections were blocked with 2% serum (of the species of the secondary antibody) in PBS, then incubated with primary antibody for 30 min at RT or overnight at 4°C followed by three washes with PBS. Sections were then incubated with biotin-conjugated secondary antibodies for 30 min at RT followed by three washes with PBS and subsequently incubated with Vectastain ABC-HRP reagent (Vector, PK-6100) for 30 minutes at RT followed by two washes with PBS. Stains were developed by applying di-aminobenzidine (DAB) substrate (Sigma, D4168) directly to the sections. Sections were counterstained with Carazzi's haematoxylin and mounted in glycerol/gelatin mounting medium (Sigma, GG1-10).

List of antibodies provided in **Supplementary Table 18.**

ELISA

The concentration of the human placental protein 14 (PP14/PAEP/glycodelin) in the organoid culture supernatants was assayed using the RayBio Human PP14 ELISA kit (RayBiotech, ELH-PP14), following manufacturer's instructions. The samples were diluted 1:2 with assay diluent and plated in duplicate. For each sample, three biological replicates were used. The emitted absorbance at 450 nm was measured with Synergy HT microplate reader (BioTek). The mean absorbance for each set of duplicate standards and samples was calculated and the average zero standard optical density was subtracted. The standard concentration and absorbance values were used for the generation of a standard curve from which sample PP14 concentrations were extrapolated.

Multiplexed smFISH and high-resolution imaging

Large tissue section staining and fluorescent imaging was conducted largely as described previously[68]. Sections were cut from FFPE blocks at a thickness of 5 μm using a microtome, placed onto SuperFrost Plus slides (VWR), and baked at 55°C to dry and ensure adhesion. Tissue sections were then processed using a Leica BOND RX to automate staining with the RNAscope Multiplex Fluorescent Reagent Kit v2 Assay (Advanced Cell Diagnostics, Bio-Techne), according to the manufacturers' instructions, in combination with immunohistochemistry (IHC)[69]. Probes described in **Supplementary Table 19**, and antibodies in **Supplementary Table 18**. Automated processing included baking at 60°C for 30 minutes and dewaxing, as well as heat-induced epitope retrieval at 95°C for 15 minutes in buffer ER2 and

digestion with Protease III for 15 minutes. Tyramide signal amplification with Opal 520, Opal 570, and Opal 650 (Akoya Biosciences) was used to develop three RNAscope probe channels. Where included, IHC was conducted after RNAscope completion. A blocking step of 1 hour in Primary Antibody Diluent (Leica) was followed by primary antibody incubation (rabbit anti-EPCAM, ab71916, or rabbit anti-FOXJ1, ab235445) at room temperature for 2 hours, and then HRP goat anti-rabbit IgG (Thermo G21234) at 1:1,500 at room temperature for 1 hour. Both antibodies were diluted in Primary Antibody Diluent. The IHC signal was developed using TSA-biotin (TSA Plus Biotin Kit, Perkin Elmer) and streptavidin-conjugated Atto 425 (Sigma Aldrich). Stained sections were imaged with a Perkin Elmer Opera Phenix High-Content Screening System, in confocal mode with 1 µm z-step size, using 20× (NA 0.16, 0.299 µm/pixel) or 40× (NA 1.1, 0.149 µm/pixel) water-immersion objectives. Channels: DAPI (excitation 375 nm, emission 435-480 nm), Atto 425 (ex. 425 nm, em. 463-501 nm), Opal 520 (ex. 488 nm, em. 500-550 nm), Opal 570 (ex. 561 nm, em. 570-630 nm), Opal 650 (ex. 640 nm, em. 650-760 nm).

10x Genomics Chromium GEX library preparation and sequencing

Cell loading and library preparation was carried out according to the manufacturer's protocol for the Chromium Single Cell 3′ Kit v.2 or v.3.0 to attain between 2,000 and 10,000 cells per well. Organoids from donors E001, B044 and B080 were multiplexed under the same reaction. Libraries were sequenced aiming at a minimum coverage of 20,000 raw reads per cell, on the Illumina HiSeq 4000 or Novaseq 6000 systems; using 26/8/0/98 cycles (read lengths) for R1/i7/i5/R2 (3' Kit v.2) or 28/8/0/91 cycles for R1/i7/i5/R2 (3' Kit v.3).

10x Genomics Visium library preparation and sequencing

Ten micron cryosections were cut and placed in duplicate on 10x Genomics Visium slides (beta product version),processed according to the manufacturer's instructions. Briefly, sections were fixed with cold methanol, stained with haematoxylin and eosin and imaged on a Hamamatsu NanoZoomer S60 before permeabilisation (endometrium 20 min), reverse transcription and cDNA synthesis using a template-switching protocol. Second-strand cDNA was liberated from the slide and single-indexed libraries prepared using a 10x Genomics PCR-based protocol. Libraries were sequenced (1 per lane on a HiSeq4000), aiming for 300M raw reads per sample using 28/8/0/91 cycles for R1/i7/i5/R2.

Alignment, quantification and donor deconvolution of scRNA-seq data

The libraries were mapped with STAR 2.7.3a, using 10x Genomics' GRCh38 reference 1.2.0 release (Ensembl 84). For single-cells samples reads were required to align within exonic regions, while for single-nuclei samples reads were required to align within whole transcript regions instead. STARsolo

CB_UMI_Simple mode was used for the alignment and quantification, with the default soloCellFilter setting of 10x Genomics' "CellRanger2.2 3000 0.99 10" emulating CellRanger 2.2's cell/soup cutoff.

Donors within the multiplexed organoid samples (E001, B044 and B080) were deconvolved using Souporcell[52]. All three-donor samples were processed individually, and the resulting donor calls produced genotype variant features that allowed the association of donor identities across samples, using Souporcell's shared sample identification.

Alignment, quantification and quality control of Visium data

Visium sequencing data were aligned and quantified using the Space Ranger Software Suite (version 1.0.0, 10x Genomics Inc) with 10x Genomics' GRCh38 reference 3.0.0 release. Spots were manually aligned to the paired H&E images by 10x Genomics. All spots under tissue were included in downstream analysis. The mean UMI counts per spot was 91283, and the mean number of genes per spot was 1962. Scanpy[48] (version 1.4.4) was used to perform downstream analysis.

Statistics and Reproducibility

No samples were excluded as part of the execution of any statistical tests. Single cells were excluded from the logistic projection if the unsupervised clustering aggregated them into groups that could be confounding the cell type identification task (e.g., low sequencing depth, predicted cell doublets and cells undergoing cell division).

The analysis of endometrial adenocarcinoma from TCGA was done on 314 samples for which the exposure obtained was above the intercept value obtained from the cellular signal analysis[61] on all 552 endometrial adenocarcinomas (n = 430 endometrioid and n = 122 serous endometrial adenocarcinomas). The association of cellular signal with cancer stage was evaluated using a Kruskal test and Wilcoxon test on the exposure value for the *LRG5+* signal. Cancer stages from 1 to 4 were regrouped into cohorts (n = 201, 26, 72 and 14 respectively); indeed, one sample was missing this annotation, so that only 313 samples were used in our statistical tests and were reported in our figures. For the Kruskal test, significant deviations in *LRG5+* exposure values were observed between cohorts ($p = 2.80E-7$). The Wilcoxon test was performed on the following binary partitions: stage 1 against state 2 to 4 combined (n = 201 and 112 respectively; $p = 3.10E-8$); stage 1 and 2 against stage 3 and 4 (n = 227 and 86 respectively; $p = 7.54E-6$); and stage 1 to 3 against stage 4 (n = 299 and 14 respectively; $p = 0.00205$). The results can be seen in Figure 3b.

The level of expression of epithelial markers within peritoneum lesions from endometriosis patients from GSE141549 was compared to that within endometrium and peritoneum from control patients. Comparisons were performed independently on donors assessed to be in proliferative and secretory phases of the menstrual cycle, and each of the three categories of peritoneum lesions were compared to both peritoneum and endometrium of control patients. For the proliferative and secretory phase comparisons, the number of independent biological samples was, respectively: control endometrium (n = 17 and n = 25), control peritoneum (n = 4 and n = 8), peritoneum red lesions (n = 2 and n = 7), peritoneum white lesions (n = 5 and n = 4) and peritoneum black lesions (n = 6 and n = 5), reported in Supplementary Table 3c. A two-sided Wilcoxon test was used to detect the significance of deviation, which was reported in Figure 3c and Extended data Figure 6b.

To correct for the variable numbers of epithelial cells from each individual, which can confound downstream analyses, we randomly downsampled 1000 epithelial cells in each individual. Differential expression (DE) analyses of epithelial cell types were performed using the Wilcoxon test and DEseq2. A lower quality cluster of epithelial cells was excluded from this analysis. All epithelial cells from the subsampled object (n = 10729) were considered for the Wilcoxon test. DEseq2 analysis excluded cells from specific donors if an insufficient number of cells (<10 cells) were aggregated into the pseudo bulk samples (see Methods). The number of independent biological samples used for DEseq2 analysis is the following: Pre-ciliated (n = 3), Ciliated (n = 10), Ciliated_LRG5 (n = 4), Glandular (n = 9), Glandular_secretory (n = 8), Lumenal_1 (n = 7), Lumenal_2 (n = 5), SOX9+LGR5- (n = 7), SOX9+LGR5+ (n = 8) and SOX9_prolif (n = 6). These results are included in Supplementary Table 3. Wilcoxon test results were used to select the relevant genes (adjusted p < 0.005 and log2 Fold Change > 0.2) for CellphoneDB

The effect of inhibitors of the NOTCH (DBZ) and WNT (XAV939) pathways on the differentiation efficiency of organoid stimulated by hormones in a three time point series was performed on three independent biological samples using scRNA-seq. The comparison of differentiation efficiencies was performed within each time point, always comparing the controls to the organoids treated with a given inhibitor (n= 3 and 3 respectively). An unpaired Z-test was performed to detect significant deviation in reported proportion between classes in relation to in-class unbiased sample variances. P values reported correspond to single-sided tests. On day 2 of the time course, the following two observations were significant when comparing the proportion of pre-ciliated cells with and without inhibitors: (i) the proportion was higher in organoids treated with NOTCH inhibitor (p = 0.01468) and, (ii) the proportion was lower in organoids treated with WNT inhibitor (p = 0.01455). On day 6, analogous observations were significant when comparing the proportion of ciliated cells with and without inhibitors: (i) the

proportion was higher in organoids treated with NOTCH inhibitor (p = 0.04159) and, (ii) the proportion was lower in organoids treated with WNT inhibitor (p = 0.00492). On day 6, two additional observations were significant when comparing the proportion of secretory cells with and without inhibitors: (i) the proportion was lower in organoids treated with NOTCH inhibitor (p = 0.03789) and, (ii) the proportion was lower in organoids treated with WNT inhibitor (p = 0.00603). These were included in Figure 7d, and Supplementary Table 11.

The effect of inhibitors of the NOTCH (DBZ, DAPT) and WNT (IWP-2, XAV939) pathways on the expression of ciliary (*PIFO*, *FOXJ1*) and secretory (*PAEP*, *SPP1*) genes in organoids stimulated by hormones in a three time point series was performed on three independent biological samples using qRT-PCR. Comparisons were performed within time points (day 2 or day 6 respectively), always comparing the controls to the organoids treated with a given inhibitor. For statistical analysis, an ordinary one-way ANOVA for unpaired and normally distributed values was used. Dunnett's test for multiple comparisons was then performed to obtain the adjusted p-values of the experimental groups against the control group (no inhibitors). Significant increase of the expression of *PAEP* is observed at day 2 after treatment with inhibitors of the WNT pathway, IWP-2 (p = 0.0009). These were included in Extended data Figure 9d.

The effect of inhibitors of the NOTCH (DBZ, DAPT) and WNT (IWP-2, XAV939) pathways on the concentration of glycodelin (PAEP) produced by organoids stimulated by hormones in a three time point series was performed on three independent biological samples using ELISA. Comparisons were performed against the controls (no hormones, no inhibitors) at day 0. For statistical analysis, an ordinary one-way ANOVA for unpaired and normally distributed values was used. Dunnett's test for multiple comparisons was then performed to obtain the adjusted p-values of the experimental groups against the control group. On day 6 there is a significant increase of the concentration of glycodelin after treatment with inhibitors of the WNT pathway, IWP-2 (p = 0.0177) and XAV939 (p = 0.0025). These were included in Extended data Figure 9e.

The number of cells overlaid in each Figure are as follows: Figure 1b-c (n = 98568), Figure 1d (n = 1850), Figure 1e and f (n = 27426), Figure 2b-c (n = 10729), Figure 6b-c (n = 37048), Figure 6f (n = 19152), Figure 6h (n = 11143), Figure 7b-c (n = 118672), Figure 7h (n = 65910). Extended data figure 1b (n = 100307), Extended data Figure 1e (n = 98568), Extended data Figure 1f (n = 7577), Extended data Figure 2b-c (n = 28498), Extended data Figure 2b-c (n = 28498), Extended data Figure 4a-b (n = 11866), Extended data Figure 8a-b (n = 37048), Extended data Figure 8c (n = 19152), Extended data Figure 8e-f (n = 37854), Extended data Figure 9b-c (n = 118638).

Staining of endometrium tissue with immunohistochemistry for KRT5, SCGB2A2, and COX-1 was performed on three proliferative endometrium and three secretory endometrium samples, each from a different donor. Representative images are shown in Figure 2h. Staining of endometrial organoids with immunohistochemistry for SCGB2A2, HEY1, FOXJ1, PAEP and acetylated-a-tubulin was performed on three biologically independent samples. Representative images are shown in Figure 6e and Figure 7e.

Assay of endometrium tissue using 10x Genomics Visium spatial transcriptomics technology was performed on one proliferative sample and one secretory sample, from different donors. Each sample was assayed in duplicate, with sections 100 µm apart. Data are shown in Figure 2e and 2g, Extended data Fig. 3a and 3d-f, and Extended data Fig. 4c-e. The analysis of gene expression on Visium slides used an unsupervised analysis to detect cell-types clusters. Proportion of mRNA originating from specific cell types was predicted by cell2location. In order to detect differentially expressed genes in epithelial cell types, the statistical test was performed on a subset of spots on Visium slides that excluded spots assessed to not contain a significant proportion of epithelial cells. The differentially expressed genes were reported in Supplementary Table 5.

LGR5, *WNT7A* and *EPCAM* transcripts, marking the *SOX9+LGR5+* population in the lumenal epithelium of proliferative endometrium, were stained by smFISH in four samples each from a different donor. One representative sample is shown in Figure 2f.

Staining of epithelial *NOTCH2* and *WNT7A* expression by smFISH and subsequent quantification by pixel classified intensity was conducted in three secretory endometrium samples each from a different donor. One representative *NOTCH2* staining image is shown in Figure 4d; quantification of expression of both *NOTCH2* and *WNT7A* in all three donors is shown plotted in Figure 4e.

Expression of the cellular proliferation marker *MKI67* in epithelia of proliferative endometrium was stained by smFISH, combined with immunohistochemistry for the epithelial marker EPCAM, was conducted in three samples each from a different donor. Images from all three donors, including representative high-magnification images of lumenal, functional, and basal regions, are shown in Extended data Figure 5a.

All histological samples used for smFISH analyses, including four proliferative endometrium samples and three secretory endometrium samples, all from different donors, were stained for cell type-specific markers by smFISH were stained with a panel of positive control genes *POLR2A*, *PPIB*, and *UBC* to

assess staining quality. Two representative donors each from the proliferative and secretory phases are shown in Extended data Figure 5b.

Coexpression of *LGR5* and FOXJ1, by combined smFISH and immunohistochemistry, was stained in two proliferative endometrium samples and two secretory endometrium samples, all from different donors. Representative images of the lumenal epithelium from all four samples are shown in Extended data Figure 5c.

Comparison of the expression of *PAEP* in epithelia of proliferative and secretory endometrium was stained by smFISH, combined with immunohistochemistry for the epithelial marker EPCAM, was conducted in two samples from each phase, all from different donors. Images from all four donors are shown in Extended data Figure 5d.

The expression of *WNT5A*, *WNT7A*, and *LGR5* in proliferative and secretory endometrium was stained by smFISH in two samples from each phase, all from different donors. Images from all four donors including high-magnification images of lumenal and glandular epithelia, are shown in Extended data Figure 7a.

Staining of the NOTCH ligand *JAG1* and downstream NOTCH target *HEY1* by smFISH was conducted in three secretory endometrium samples each from a different donor. Representative images of all three donors comparing *JAG1* expression in lumenal and glandular epithelia are shown in Extended data Figure 7b; high-magnification images of *JAG1-HEY1* coexpressing and juxtacrine cells are shown in Extended data Figure 7c.

Supplementary references

67. Roberts, K. & Tuck, L. Embedding and freezing fresh human tissue in OCT using isopentane V.3. *protocols.io* (2019) doi:10.17504/protocols.io.95mh846.

68. Bayraktar, O. A. *et al.* Astrocyte layers in the mammalian cerebral cortex revealed by a single-cell in situ transcriptomic map. *Nat. Neurosci.* (2020) doi:10.1038/s41593-020-0602-1.

69. Roberts, K. & Bayraktar, O. A. Automation of Multiplexed RNAscope Single-Molecule Fluorescent In Situ Hybridization and Immunohistochemistry for Spatial Tissue Mapping. in *In Situ Hybridization Protocols* (eds. Nielsen, B. S. & Jones, J.) 229–244 (Springer US, 2020).
