# Supplementary material for: Mapping the temporal and spatial dynamics of the human endometrium in vivo and in vitro
Source: Nat Genet. 2021 Dec 2;53(12):1698–711. doi: 10.1038/s41588-021-00972-2 (PMC8648563; doi:10.1038/s41588-021-00972-2)
Supplement: Supplementary file 2 — Reporting Summary [file 41588_2021_972_MOESM2_ESM.pdf]

nature research

Corresponding author(s): Roser Vento-Tormo

Last updated by author(s): Sep 13, 2021

Reporting Summary

Nature Research wishes to improve the reproducibility of the work that we publish. This form provides structure for consistency and transparency in reporting. For further information on Nature Research policies, see our Editorial Policies and the Editorial Policy Checklist.

Statistics

For all statistical analyses, confirm that the following items are present in the figure legend, table legend, main text, or Methods section.

n/a	Confirmed	
☐	☒	The exact sample size (*n*) for each experimental group/condition, given as a discrete number and unit of measurement
☐	☒	A statement on whether measurements were taken from distinct samples or whether the same sample was measured repeatedly
☐	☒	The statistical test(s) used AND whether they are one- or two-sided<br>*Only common tests should be described solely by name; describe more complex techniques in the Methods section.*
☐	☒	A description of all covariates tested
☐	☒	A description of any assumptions or corrections, such as tests of normality and adjustment for multiple comparisons
☐	☒	A full description of the statistical parameters including central tendency (e.g. means) or other basic estimates (e.g. regression coefficient) AND variation (e.g. standard deviation) or associated estimates of uncertainty (e.g. confidence intervals)
☐	☒	For null hypothesis testing, the test statistic (e.g. F, t, r) with confidence intervals, effect sizes, degrees of freedom and P value noted<br>*Give P values as exact values whenever suitable.*
☒	☐	For Bayesian analysis, information on the choice of priors and Markov chain Monte Carlo settings
☒	☐	For hierarchical and complex designs, identification of the appropriate level for tests and full reporting of outcomes
☒	☐	Estimates of effect sizes (e.g. Cohen's *d*, Pearson's *r*), indicating how they were calculated

Our web collection on statistics for biologists contains articles on many of the points above.

Software and code

Policy information about availability of computer code

Data collection	Perkin Elmer Harmony v4.9; BIOP Perkin Elmer Acapella Stitcher; Chromium Controller Firmware version 4.00 (10X genomics)
Data analysis	o Alignment, quantification and donor deconvolution of scRNA-seq data: STAR 2.7.3a (with 10x Genomics' GRCh38 reference 1.2.0 release); Souporcell.<br>o Alignment, quantification and quality control of Visium data: Space Ranger Software Ranger version 1.0.0 (with 10x GRCh38 reference 3.0.0 release); Scanpy (version 1.4.4)<br>o Downstream scRNA-seq and Visium analysis: Scanpy (version 1.4.4); Scrublet (version 0.2.1) ; scVI (version 0.6.5); decontX (under the "celda" R package version 1.5.11); Seurat (version 3.2.1) Palantir (version 1.0.0); cell2location (version 0.5-alpha); Dorothea (version 1.0); edgeR (version 3.32.1) and limma (version 3.46.0).<br>o qPCR & ELISA: Graphpad Prism version 9<br>o Image analysis: Ilastik (version 1.3.3) and Napari (version 0.4.2).<br>o Custom code available at https://github.com/Ventolab/UHCA and https://github.com/Ventolab/CellphoneDB

For manuscripts utilizing custom algorithms or software that are central to the research but not yet described in published literature, software must be made available to editors and reviewers. We strongly encourage code deposition in a community repository (e.g. GitHub). See the Nature Research guidelines for submitting code & software for further information.

April 2020

Data

Policy information about availability of data

All manuscripts must include a data availability statement. This statement should provide the following information, where applicable:

- Accession codes, unique identifiers, or web links for publicly available datasets
- A list of figures that have associated raw data
- A description of any restrictions on data availability

Datasets generated for this manuscript were uploaded into ArrayExpress under accession number E-MTAB-10287 (scRNA-seq in vivo); E-MTAB-9260 (Visium in vivo) and E-MTAB-10283 (scRNA-seq in vitro). snRNA-seq in vivo data was uploaded into the European Genome Archive; EGAD00001007909 (controlled access). Controlled access for the human samples used for snRNA-seq is requested by the patient consent form. Request for data access will be referred directly to the Data Access Committee of this work: datasharing [at] sanger [dot] ac [dot] uk. The approximate timeframe for external researchers to access the data after permission would be around 2-3 weeks.

Tumor bulk transcriptomes for endometrioid (430 samples) and serous (112 samples) endometrial adenocarcinoma were downloaded from The Cancer Genome Atlas (TCGA).

Additional single-cell transcriptomes of 10 endometrial biopsies from PMID: 32929266 were downloaded from GEO with GSE111976.

Processed matrices can be accessed and downloaded from www.reproductivecellatlas.org.

Image datasets were deposited into the EMBL-EBI BioImage Archive under accession S-BIAD190

Field-specific reporting

Please select the one below that is the best fit for your research. If you are not sure, read the appropriate sections before making your selection.

- [x] Life sciences
- [ ] Behavioural & social sciences
- [ ] Ecological, evolutionary & environmental sciences

For a reference copy of the document with all sections, see nature.com/documents/nr-reporting-summary-flat.pdf

Life sciences study design

All studies must disclose on these points even when the disclosure is negative.

Sample size

No sample-size calculation was done due to the nature of the study. We collected endometrial biopsies from live donors (n=3) and full-thickness uterine tissue from donors who died of non-gynaecological causes (n=6). Based on previous single-cell transcriptomics atlases (PMID: 30429548; PMID: 31209336; PMID: 32066951), the size cohort should be sufficient to capture the main cell types in a tissue. In addition, we integrated our data with publicly available single-cell transcriptomes of 10 endometrial biopsies (PMID: 32929266). The integration with publicly available datasets allowed us to confirm the endometrial subsets defined using our cohort, thus, confirming the appropriate sample size considered.

Data exclusions

Two of the uterine samples profiled (individuals A10, A16) were not included in the final dataset. The analysis of these two samples collected from organs donors showed the presence of abnormal epithelial subsets, and thus, the samples were not considered "healthy".
For the final count matrix, we excluded cells based on pre-established criteria for single-cells: we excluded low quality samples and contaminating cells (i.e.- cells with low number of detected genes, high mitochondrial content or cells detected as doublets).

Replication

Data was integrated with 10 additional endometrial biopsies (PMID: 32929266) and same endometrial populations were shown on these datasets. In addition, results were validated using orthogonal methods, such as immunohistochemistry and/or smFISH. All attempts at replication were successful.

Randomization

Only healthy individuals were considered in our analysis. Randomisation was not relevant due to the study design do not involve healthy vs disease comparisons.

Blinding

Only healthy individuals were considered in our analysis. Blinding was not relevant due to the study design do not involve healthy vs disease comparisons.

Reporting for specific materials, systems and methods

We require information from authors about some types of materials, experimental systems and methods used in many studies. Here, indicate whether each material, system or method listed is relevant to your study. If you are not sure if a list item applies to your research, read the appropriate section before selecting a response.

Materials & experimental systems

n/a	Involved in the study
☐	☒ Antibodies
☒	☐ Eukaryotic cell lines
☒	☐ Palaeontology and archaeology
☒	☐ Animals and other organisms
☐	☒ Human research participants
☒	☐ Clinical data
☒	☐ Dual use research of concern

Methods

n/a	Involved in the study
☒	☐ ChIP-seq
☒	☐ Flow cytometry
☒	☐ MRI-based neuroimaging

Antibodies

Antibodies used

Primary Antibodies:
Target | Clone | Product number | Supplier | Host species | Clonality | Dilution | Antigen retrieval buffer
EPCAM - ab71916 Abcam Rabbit Polyclonal 1:1,500 BOND ER2, pH 9.0
FOXJ1 EPR21874 ab235445 Abcam Rabbit Monoclonal 1:1,500 BOND ER2, pH 9.0
Acetyl-alpha-Tubulin (Lys40) 6-11B-1 12152S Cell Signalling Technologies Mouse Monoclonal 1:1000, 1:100 AR pH 9.5 buffer
FOXJ1 - HPA005714 Atlas Rabbit Polyclonal 0.215277778 AR pH 6.4 buffer
SCGB2A2 EPR9092 ab150359 Abcam Rabbit Monoclonal 0.215277778 AR pH 9.5 buffer
HEY1 - ab22614 Abcam Rabbit Polyclonal 01:50 AR pH 9.5 buffer
Glycodelin (PAEP) EP870Y ab 53289 Abcam Rabbit Monoclonal 1:500, 1:100 AR pH 9.5 buffer
KRT5 EP1601Y ab52635 Abcam Rabbit Monoclonal 0.145833333 AR pH 9.5 buffer
COX-1 EPR5866 ab109025 Abcam Rabbit Monoclonal 0.458333333 AR pH 6 buffer

Secondary antibodies:
Target | Conjugate | Product number | Supplier | Host species | Clonality | Dilution
Rabbit IgG HRP G21234 Thermo Fisher Goat Polyclonal 1:1,500
Rabbit IgG Biotin BA-1000 Vector Goat Polyclonal 0.180555556
Mouse IgG Biotin BA-2000 Vector Horse Polyclonal 0.152777778

Validation

Anti-EPCAM ab71916 and anti-FOXJ1 ab235445 were considered validated based upon:
- Staining gave the expected patterns (EPCAM: as a canonical marker of epithelial cells, matching the morphology identified on H&E staining of adjacent sections; FOXJ1: as a canonical nuclear-enriched transcription factor protein)
- Antibodies were co-stained with RNAscope against the RNA transcript of the same targets, and gave matching results.
- Staining strongly resembled the manufacturer's example images.

For all the others (except COX-1):
- Staining showed the expected pattern (ac-a-tub positive in cilia/FOXJ1 in nuclei of ciliated cells as it is a transcription factor, HEY1, KRT5, SCGB2A2, PAEP in glandular or surface cells)
- Stainings were considered true when compared to a negative control (for negative control the primary antibody was replaced by equivalent species IgG antibodies)
- Staining strongly resembled the manufacturer's example images.

For COX-1 staining:
The antibody data sheet recommends 1/150 with heat treatment at 98C in TRIS buffer which produces very limited staining of just the surface epithelium. However, at 125C in TRIS buffer there is much clearer staining of the surface epithelium with more positive cells but also staining of some cells within glands. We therefore selected images achieved using this condition. Antibody dilution was 1/800.

Human research participants

Policy information about studies involving human research participants

Population characteristics

Tissues were collected from 9 individuals from the United Kingdom (biopsies and full thickness uterine wall samples A13 & A30) and United States (Trv2, Trv3, Trv4 and Trv5). All women that participated in our study were in reproductive age.

Recruitment

All women that participated in our study were in reproductive age and women recruited for the endometrial biopsy collection were not under hormonal treatment for at least 3 months, and this was the main recruitment criteria used to include individuals in our study. We believe this method of recruitment does not represent any bias that can impact our result.

Ethics oversight

Full thickness uterine wall samples (A13, A30) were obtained from deceased transplant organ donors after ethical approval (reference 15/EE/0152, East of England-Cambridge South Research Ethics Committee). Additional full thickness uterine wall samples were collected from four women during autopsy (Trv2, Trv3, Trv4 and Trv5). The use of these tissues was approved by the London, Surrey Research Ethics Committee (REC reference 17/LO/1801, 26/10/2017). In both cases, informed consent was obtained from the legally recognised next-of-kin.

Endometrial biopsies were obtained from Newcastle Upon Tyne Hospitals after ethical approval (reference 16/NE/0167,

North East - Newcastle & North Tyneside 1 Research Ethics Committee), Addenbrooke's Hospital under ethical approval from the East of England-Cambridge South Research Ethics Committee (08/H0305/40); and Bourne Hall Clinic under ethical approval from the East of England-Central Research Ethics Committee for the 'Biology of the Human Uterus in Pregnancy and Disease Tissue Bank' run by the Centre for Trophoblast Research (17/EE/0151). Endometrial biopsies were obtained from live donors with written informed consent from all participants.

Note that full information on the approval of the study protocol must also be provided in the manuscript.
